# RNA-Seq analysis reveals functionally relevant coding and non-coding RNAs in crossbred bull spermatozoa

**DOI:** 10.1016/j.anireprosci.2020.106621

**Published:** 2020-11

**Authors:** Mani Arul Prakash, Arumugam Kumaresan, Manish Kumar Sinha, Elango Kamaraj, Tushar Kumar Mohanty, Tirtha Kumar Datta, Jane M. Morrell

**Affiliations:** a Theriogenology Laboratory, Southern Regional Station of ICAR-National Dairy Research Institute, Adugodi, Bengaluru, 560030 Karnataka, India; b Animal Reproduction, Gynaecology and Obstetrics, National Dairy Research Institute, Karnal, 132001 Haryana, India; c Animal Biotechnology Centre, National Dairy Research Institute, Karnal, 132001 Haryana, India; d Clinical Sciences, Swedish University of Agricultural Sciences, 750 07 Uppsala, Sweden

**Keywords:** Crossbred Bull, Spermatozoa, Transcriptomic profiling, RNA-Seq, Ribosome, Oxidative phosphorylation

## Abstract

Sperm, which are believed to be transcriptionally and translationally inactive, synthesize RNA and proteins before there is gradual disappearance of the ribosome during chromatin compaction. Sperm transfer several functionally relevant transcripts to the oocyte, controlling maternal-zygotic transition and embryonic development. The present study was undertaken to profile and analyze sperm transcripts comprehensively using Next Generation Ribonucleic acid sequencing technology in Holstein Friesian x Tharparkar crossbred bulls. The results from global transcriptomic profiling revealed transcripts for 13,814 genes; of which 431 transcripts were expressed with >1 FPKM and 13,383 transcripts were expressed with >0 or <1 FPKM. The abundant mRNA transcripts of crossbred bull sperm were *PRM1* and *HMGB4*. Gene ontology of transcripts with>1 FPKM revealed there was a major involvement in the structural constituent of ribosomes and translation. Results from pathway enrichment indicated the connection between ribosome, oxidative phosphorylation and spliceosome pathways and the transcripts of crossbred bull spermatozoa. The transcriptional abundance of selected genes, validated using RT-qPCR, indicated significant variations between bulls. Collectively, it may be inferred that the transcripts in crossbred bull sperm were heavily implicated in functions such as the structural constituent of ribosomes and translation, and pathways such as ribosome, oxidative phosphorylation and spliceosome. Further studies using larger sample sizes are required to understand the possible implications of transcriptomic variations on semen quality and fertility.

## Highlights

• RNA-Seq analysis was done to characterize the transcriptome of crossbred bull spermatozoa.
• Among the 13,814 transcripts detected, 431 had FPKM > 1 and 13,673 had FPKM > 0 or < 1.
• Coding and non-coding RNAs account for 13,145 (95.15%) and 152 (1.1%), respectively.
• Sperm transcripts were mainly related to ribosome, oxidative phosphorylation and spliceosome pathways.
• qPCR analysis showed individual variations in transcriptional abundance of selected genes.

## 1 Introduction

Spermiogenesis involves a myriad of intricate processes that include the replacement of histones by transition proteins, which will, in turn, be replaced by protamine to form compact condensed chromatin. Throughout this process, cytoplasm and ribosomes gradually disappear, resulting in the transcriptionally restricted mature sperm (Jodar and Oliva, 2014). Occasional reports of RNA synthesis in sperm were considered to arise from contamination by somatic cells or the presence of immature spermatids in samples (Miller and Ostermeier, 2006). Others, however, reported the translation of proteins by mitochondrial ribosomes during sperm capacitation and expression during sperm transit of the female reproductive tract prior to fertilization (Zhao et al., 2009). Hence, there are inconsistencies in thought regarding possible transcription and translation processes in the mammalian male gamete (Ren et al., 2017). To mitigate these intricacies and to understand sperm mRNA transcript differences in populations, several studies have been conducted using techniques such as suppression subtractive hybridization (Chen et al., 2014), microarrays (Suliman et al., 2018) and single cell RNA sequencing (Tomoiaga et al., 2020). Next generation sequencing (NGS) of sperm RNA is the current gold-standard technology for analysis of global/complete transcriptome profiles to understand the molecular basis of an individual (Wang et al., 2009). Sperm transcriptome studies using RNA sequencing were conducted earlier in several species, such as humans (Sendler et al., 2013), bulls (Card et al., 2017; Selvaraju et al., 2017; Singh et al., 2019; Raval et al., 2019), boars (Yang et al., 2009), stallions (Das et al., 2013) and laboratory animals (Margolin et al., 2014).

Results from transcriptomic profiling of sperm RNA has provided evidence for a wide variety of RNA molecules which may have important functions in sperm development, chromatin repackaging (Hamatani, 2012), genomic imprinting, zygote development **(**Das et al., 2013), and post-fertilization events (Selvaraju et al., 2018). Spermatozoal mRNA transcripts form an integral part of many processes, such as genome recognition, consolidation-confrontation, early embryonic development (Sendler et al., 2013), epigenetic trans-generation inherence (Rando, 2016) and placental development (Selvaraju et al., 2018). Furthermore, an association of sperm transcripts with male fertility has also been discovered (Selvaraju et al., 2018; Bianchi et al., 2018) and suggested as a fertility prediction approach (Jodar et al., 2013; Card et al., 2017; Selvaraju et al., 2017); however, predictive accuracies have proved to be variable. Breed variations in sperm transcriptomic profile have also been reported (Raval et al., 2019; Singh et al., 2019).

It is now understood that the incidence of infertility/sub-fertility is greater in crossbred bulls compared to either exotic purebred or zebu bulls (Vijetha et al., 2014; Tripathi et al., 2014; Aslam et al., 2015; Vignesh et al., 2020; Elango et al., 2020). Results from recent studies indicated there is variation in the sperm mRNA transcripts between and within different species/breeds such as *Bos taurus* and *Bos indicus* (Selvaraju et al., 2017; Raval et al., 2019; Singh et al., 2019). Thus, sperm transcriptomic data of *Bos taurus* or *Bos indicus* cattle may not be useful to predict the transcriptome and its function in crossbred (*Bos taurus* × *Bos indicus*) bull spermatozoa, because crossbred cattle result from RNA-RNA amalgamation between the gametes of two different breeds during fertilization (Jodar et al., 2013). Possible variation of transcripts in crossbred bull spermatozoa, therefore, is to be expected and is hitherto unexplored. In the present study, using Next Generation Ribonucleic Acid Sequencing technology, there was transcriptomic profiling of Holstein Friesian crossbred bull spermatozoa.

## 2 Materials and methods

The present study was conducted at the Theriogenology laboratory, ICAR - National Dairy Research Institute (NDRI), Karnal. The study protocol was duly approved by the Institute Animal Ethics Committee (IAEC/4/36) and performed in accordance with relevant guidelines and regulations. Cryopreserved semen from six Holstein Friesian crossbred (Holstein Friesian X Tharparkar) breeding bulls, routinely used for artificial breeding, were procured from the Artificial Breeding Research Centre of NDRI, Karnal, Haryana, India. Frozen-thawed spermatozoa from four of these bulls were utilized for global transcriptomic analysis. Frozen semen straws prepared from three ejaculates from each bull were pooled to create representative sperm samples for individual bulls. Thus, a total of four individual frozen-thawed sperm samples (from four bulls) were used for transcriptomic analysis. Validation of transcriptional abundance of selected genes were conducted using frozen-thawed spermatozoa from all the six bulls.

The age of the experimental bulls ranged between 4 and 6 years. All the bulls were maintained using similar management practices. Vaccination, deworming, regular check-up for communicable diseases, and other herd-health programs were imposed as per the farm schedule, and the bulls were free from major infectious diseases. Ejaculates were collected using the artificial vagina (IMV Technologies, France) method twice a week by allowing the bull to mount over a male “teaser” bull. After initial semen evaluation, the ejaculates were extended in Tris-egg yolk–citrate extender at the concentration of 80 million per ml, filled in 0.25-mL French straws (each straw containing 20 million spermatozoa) and equilibrated at 4 °C for 4 h before placing the samples in liquid nitrogen vapor for 10 min. The straws were subsequently plunged into liquid nitrogen for frozen storage.

### 2.1 Sperm RNA isolation

Sperm total RNA was isolated using TRIzol (Ambion, Thermo Fisher Scientific, USA) as previously described by Parthipan et al. (2015) with minor modifications. In brief, frozen semen straws from experimental bulls (60 × 10^6^ spermatozoa/bull) were thawed in a 37 °C water bath for 30 s. The contents were pooled, centrifuged at 800 x *g* for 10 min at 4 °C, the supernatant was removed without disrupting the consistency of the sperm pellet and the pellet was washed twice with 1 mL of DPBS at 800 x *g* for 15 min at 4 °C. The sperm pellet was purified by mixing with 1 mL of somatic cell lysis buffer (0.1 % SDS, 0.5 % Triton X-100 in DEPC treated water). The pellet was then placed on ice for 1 h with regular vortexing every 15 min and centrifuged at 5939 x *g* for 1 h at 4 °C. The pellet was then dissolved in 1 mL DEPC treated water and centrifuged at 5939 x *g* for 15 min at 4 °C. The washed sperm pellet was suspended in 1 mL pre-warmed TRIzol reagent (65 °C) and the sperm were shredded by sonication for 1 min and 30 s (shredding for 6 s and break for 5 s). The samples were then vortexed for 5 min and incubated for 1 h in a dry bath (62 °C) for complete dissociation of the sperm membrane. To the lysate, 200 μL of chloroform was added, the sample was mixed vigorously for 20 s and allowed to stand without disturbance at room temperature for 15 min. The mixture was centrifuged (13,362 x *g*, 4 °C, 20 min) without brake to separate the phases. Following centrifugation, there were three layers, the clear aqueous layer containing RNA, the opaque white interphase containing DNA and the red bottom organic layer containing protein. The upper aqueous layer containing RNA was carefully aspirated without disturbing the interphase and transferred to another tube. To this solution, isopropanol (0.5 mL) was added and gently mixed by inverting the tubes. The mixture was then incubated at room temperature for 15 min and centrifuged at 13,362 x *g* for 15 min at 4 °C. The supernatant was discarded, and 1 mL of 70 % ethanol was added to the RNA pellet for centrifugation at 13,362 x *g* for 10 min. The RNA pellet was air-dried to remove traces of ethanol and dissolved in 20–40 μl of DEPC-treated water for RNA quantification using a Nanodrop (ND-1000, Thermo-scientific, USA). The RNA samples with 260/280 ratio of 1.7–2.0 were processed immediately for cDNA synthesis, followed by storage at −20 °C until further processing.

### 2.2 Transcriptome library preparation

Total RNA (1 μg) was used to enrich mRNA using NEB Magnetic mRNA Isolation Kit (Illumina, USA). The transcriptome library was prepared using NEB ultra II RNA library prep kit (Illumina, USA) and sequenced using Illumina Next Seq 500 (Illumina, USA) paired end technology. The enriched mRNA was fragmented (approximately 200 bp) using fragmentation buffer. Random hexamer primers were then added and hybridized to complementary RNA sequences. These short fragments were used as templates to synthesize the first-strand of cDNA using reverse transcriptase and dNTP. The DNA-RNA hybrids produced during first strand cDNA synthesis are converted into full-length double-stranded cDNAs using RNase H and *E. coli* DNA polymerase I. The second strand of cDNA was synthesized using second strand enzyme mix and buffer. The double-stranded cDNA fragments were purified using 1.8X Ampure beads and end repaired to ensure that each molecule was free of overhangs and had 5′ phosphates and 3′ hydroxyls before the adaptor ligation. The adaptor ligated DNA was then purified using Ampure beads and enriched with specific primers, compatible for sequencing on to the Illumina platforms. The final enriched library was purified and quantified by Qubit® Fluorometer and the size was analyzed using a Bio-analyzer.

### 2.3 RNA sequencing and bioinformatics analysis

The cDNA synthesized sperm samples from four bulls were sequenced using Illumina Nextseq-500 sequencing system (Sandor® Lifesciences Pvt. Ltd. Banjara Hills, Hyderabad, India) to generate paired-end 76bp reads. The sequence analysis was conducted using online server tool Galaxy (https://usegalaxy.org/). The raw data generated from the four samples were evaluated for the read quality using *FastQC* (Galaxy version 0.72) program and the reads were processed with *Cutadapt* tool (Galaxy Version 1.18). Processing includes removal of adapter (AGATCGGAAGA) sequence, length trimming (>15bp) and quality trimming (30 phreds score). All the processed reads were then pooled and aligned to the bovine genome (*Bos taurus* UMD 3.1.94/Btau8) using *HISAT2* (Galaxy Version 2.1.0+galaxy3) and the sample was sorted with aligned sequences using Samtools (Galaxy Version 2.0.2). The mapped and properly paired sequence to the reference genome was calculated based on tabular descriptive statistics dataset tool *Flagstat* (Galaxy Version 2.0.1). With the *Cufflink* tool (Galaxy Version 2.2.1.2) the presence of individual transcripts and the mRNA transcript abundances were expressed as fragments per kilobase of exon per million fragments mapped (FPKM). The depth of coverage of transcripts was analyzed using *Genome Analysis Toolkit (GATK)* (Galaxy Version 0.0.2). Based on the FPKM values the transcripts were classified as >1 and <1 or >0 and subjected to gene ontology (GO) analysis, pathway enrichment and network analysis.

### 2.4 Gene ontology, pathway and network analysis

Gene ontology analysis and functional annotation of individual transcripts were analyzed using *Uniport* (https://www.uniprot.org/) and *The Database for Annotation, Visualization and Integrated Discovery (DAVID)* Bioinformatics Resources v6.8 (https://david.ncifcrf.gov/) with three main categories such as molecular function (MF), biological process (BP), cellular component (CC) and Kyoto Encyclopedia of Genes and Genomes (KEGG) pathway analysis. The top ten biological processes, cellular component and molecular function were plotted as a Donut pie chart using *Highcharts* (https://www.highcharts.com/demo/pie-donut). Pathway enrichment was conducted using clusterProfiler and enrich KEGG function. Interaction of genes and detailed network analysis of combined GO categories and pathway analysis were performed using *ClueGo* (version 2.5.4) and *Cluepedia* (version 1.5.4) plugins in the open source *Cytoscape* (version 3.7.1) platform (Locatelli et al., 2019). All the analyses were performed with *Bos taurus* genome as background.

### 2.5 Validation of mRNA transcript abundances using quantitative Real-Time PCR

There were a few genes (*TNP2, TNP1, RACK1, TSSK6, RASSF3, IQCF1 and ZNF706)* selected based on FPKM and functional relevance to spermatogenesis, sperm function and fertilizing potential, and were validated using quantitative Real-Time PCR in spermatozoa from six bulls. The annealing temperatures of primers for the selected genes were optimized using PCR (Prima-96 plus, Himedia). The primer sequence, product size and annealing temperatures are given in Table 1. The cDNA prepared from different bull semen samples was subjected to quantitative Real-Time PCR experiment using Insta Q96 Plus Real Time Machine PCR system (HiMedia, India). Relative abundances of mRNA transcripts were normalized to *GAPDH* abundance using the ΔCt method. Each reaction was performed with a 20 μL reaction comprising 2 μL cDNA, 0.5 μL (10 pmol/ μL) forward and reverse primers, and 10 μL of Maxima SYBR Green/ROX qPCR master mix 2 × . The thermal cycling conditions consisted of initial denaturation at 95 °C for 10 min, followed by 40 cycles of 95 °C for 15 s, 60 °C for 30 s, and 72 °C for 30 s. and all the reactions were performed in duplicates.

**Table 1:** Primers used for Real time expression quantification.

S. No	Genes	Primer sequence	Product size	Annealing temperature	Accession number
1.	*TNP2*	FP-ACAGACACACCATGCACTCC	172 bp	60 °C	NM_174200.1
		RP-TCAGTTGTACTTCCGTCCTGAG			
2.	*TNP1*	FP-TGTCGACCAGCCGCAAATTA	149 bp	60 °C	NM_174199.2
		RP-ATTGCGATTGGCATCGTCAC			
3.	*RACK1*	FP-ATCTGGGACTTGGAGGGCAA	172 bp	60 °C	NM_175802.3
		RP- CGATGGTTACCTGCCACACT			
4.	*TSSK6*	FP- CGACCTCAAGTGCGAAAACG	107 bp	60 °C	XM_027548402.1
		RP- TGGTGCTCAGATCGGGGTAT			
5.	*RASSF3*	FP- GAAGTGATTGAGGCCCTGCT	107 bp	60 °C	NM_001192886.2
		RF- AGCTTGCAGGCGTAGACTTG			
6.	*IQCF1*	FP- AGGCAGAGGCTCAACAAGAG	116 bp	60 °C	NM_001075773.1
		RF- TGCTTCTGGATCACTGACGG			
7.	*ZNF706*	FP-TGAGAGAACAAAGGCTGCGAG	181 bp	60 °C	NM_001199073.1
		RP-GGGTCTGGCATTTGTGTCCTA			
8.	*GAPDH*	FP- CTGAGGACCAGGTTGTCTCCTG	141 bp	60 °C	NM_001034034.1
		RP- CCCTGTTGCTGTAGCCAAATTC			

### 2.6 Statistical analysis

For the transcriptomic raw data analysis, the processed reads were mapped to the reference genome. The relative abundances of mRNA transcripts were analyzed using the number of reads getting mapped to the exonic region. Based on the number of reads mapped, the “normalized expression” was calculated in FPKM (fragments per kilobase of transcript model per million reads mapped). Functional enrichment analyses of gene ontology terms and KEGG pathway terms were conducted using DAVID. Based on the input list of genes and genes involved in a particular pathway, Fisher Exact *P*-value was used to calculate the EASE score, which provides a significantly enriched term. The EASE score cut-off was given by *P*-value < 0.01. Thus, the terms with *P*-values of less than 0.01 were obtained as the enriched term after enrichment analysis. The minimum count of genes considered for the term association was > = 2. False discovery rate, the corrected *P*-value, was obtained by Multiple corrections using Benjamini and Bonferroni testing. With qPCR, the transcript abundance (2^−ΔCt^) of genes was calculated after normalizing the transcript abundance to the abundance of mRNA for the housekeeping gene (*GAPDH*). The Wilcoxon signed rank test was used to compare the relative abundances of mRNA transcripts with the hypothetical median. All statistical analyses were conducted using the SPSS for Windows software (version 22, SPSS, Inc.) and Excel software (Microsoft Corp.).

## 3 Results

Results from the bioanalyzer analysis of sperm RNA revealed that it was devoid of 18 s and 28 s rRNA peaks, indicating that the total RNA isolated had no contamination of somatic cells, leucocytes, testicular germ cell and other cells. The raw and processed reads were 116,982,528 (116 million) and 98,324,960 (98 million), respectively with paired end 76 bp reads. The processed reads were properly mapped (55.1 %) and properly paired (45.6 %) with the reference bovine *Bos taurus* genome.

### 3.1 Global transcriptomic profile of crossbred spermatozoa

**Fig. 1:**
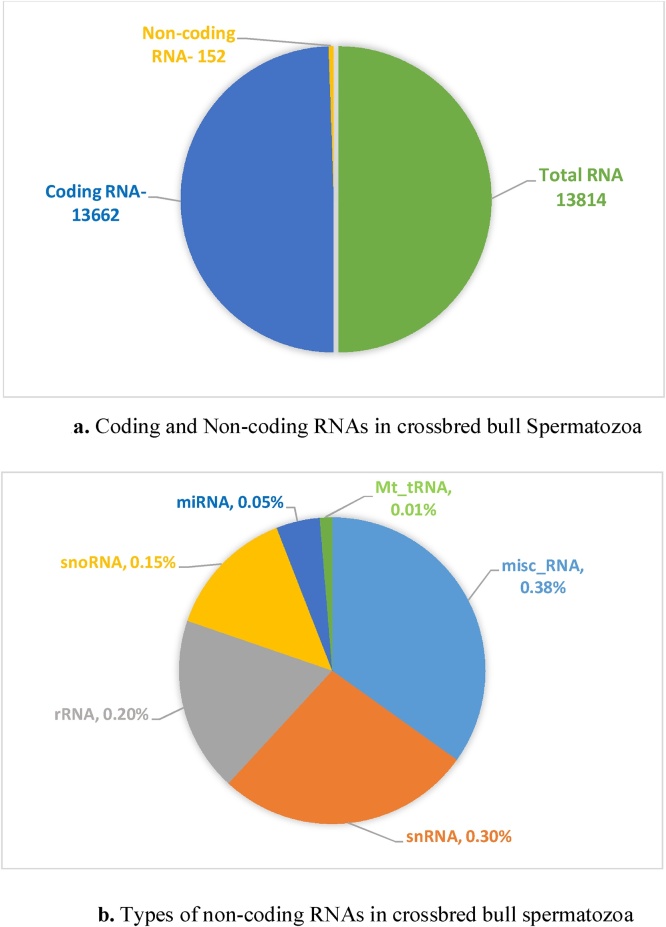
a) Coding and Non-coding RNAs in crossbred bull Spermatozoa. b) Types of non-coding RNAs in crossbred bull spermatozoa.

A total of 13,814 transcripts were identified in the crossbred bull spermatozoa, among which, 16 transcripts (non-coding RNAs) had >100 Fragments Per Kilobase of transcript per Million mapped reads (FPKM); 74 transcripts (four protein coding, two pseudogenes and 68 non-coding RNAs) had FPKM > 10; 106 transcripts (20 protein coding transcripts, three pseudogenes, 83 non-coding RNAs) had FPKM > 5; 141 transcripts (47 protein-coding transcripts, four pseudogenes, 90 non-coding RNAs) had FPKM > 3 and 431 transcripts (292 protein-coding, 22 pseudogenes, five processed pseudogenes, 112 non-coding RNAs) had FPKM > 1. Coding and non-coding RNAs and the types of RNAs in crossbred bull spermatozoa are depicted in Fig. 1ab. Among the total of 13,814 sperm transcripts, 891 were of full length with >80 % depth of coverage, in which 606 were protein-coding, 117 were non-coding RNA, 130 were pseudogenes and 38 were processed pseudogenes. (Suppl. file Excel-1). A downstream analysis of the 431 transcripts with FPKM > 1 revealed 254 (58.9 %) categorized, 38 (8.8 %) uncategorized protein-coding RNA, and 112 (25.98 %) non-coding RNAs. These non-coding RNAs included miscellaneous RNAs (misc_RNA) - 4.6 %, small nuclear RNA (snRNA) - 8.8 %, small nucleolar RNAs (snoRNA) - 4.9 %, ribosomal RNA (rRNA) - 5.6 %, micro RNA (miRNA) - 1.6 % and mitochondrial ribosomal RNA (Mt_rRNA) - 0.46 %. There were 22 (5.1 %) and five (1.2 %) Ensembl biotypes - pseudogenes and processed pseudogenes, respectively. The top ten transcripts expressed with FPKM > 1 were mostly non-coding RNAs such as rRNA, miRNA and misc_RNA (Table 2). After excluding non-coding RNAs, pseudogenes and processed pseudogenes, the top ten transcript (FPKM > 1) codes for protein are provided in Table 3.

**Table 2:** Top ten transcripts based on FPKM.

SI. No	Transcript ID	Gene Symbol	Gene Name	FPKM
1	ENSBTAT00000065080*	RF00002	rRNA	4107.9
2	ENSBTAT00000065688*	RF00002	rRNA	3444.4
3	ENSBTAT00000064298*	RF00001	rRNA	1019.3
4	ENSBTAT00000064151*	RF00001	rRNA	1007.1
5	ENSBTAT00000066008	RF00001	rRNA	735.6
6	ENSBTAT00000066086	ENSBTAG00000047329	miRNA	395.7
7	ENSBTAT00000040824*	RF00001	rRNA	276.5
8	ENSBTAT00000064653*	RF00017	misc_RNA	204.1
9	ENSBTAT00000051821	ENSBTAG00000047010	miRNA	194.8
10	ENSBTAT00000064351*	RF00017	misc_RNA	174.8

* Full length transcripts (>80 %).

**Table 3:** Top ten coding RNA, based on FPKM (>1 FPKM).

SI. No	Transcript ID	Gene Symbol	Gene Name	FPKM
1	ENSBTAT00000046692*	*PRM1*	Protamine 1	47.2
2	ENSBTAT00000000438*	*HMGB4*	High mobility group protein B4	12.1
3	ENSBTAT00000027004	*CCDC181*	Coiled-coil domain containing 181	11.2
4	ENSBTAT00000016431	*CHMP5*	Charged multivesicular body protein 5	10.7
5	ENSBTAT00000054025*	*TNP2*	Nuclear transition protein 2	8.9
6	ENSBTAT00000003201	*RPS28*	40S ribosomal protein S28	8.2
7	ENSBTAT00000006781	*RPL37*	60S ribosomal protein L37	8.1
8	ENSBTAT00000013402	*TPT1*	Translationally-controlled tumor protein	7.6
9	ENSBTAT00000064378	*ENSBTAG00000047411*	Uncharacterized protein	7.6
10	ENSBTAT00000044628	*ANKRD9*	Ankyrin repeat domain 9	7.4

* Full length transcripts (>80 %).

### 3.2 Gene ontology (GO) analysis

**Fig. 2:**
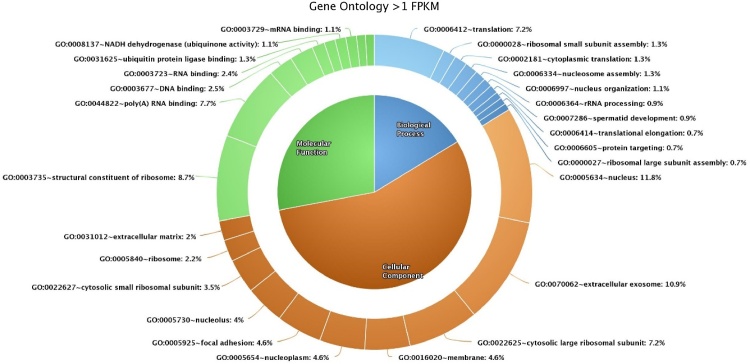
Top ten gene ontology (biological process, cellular component, molecular function) categories of transcripts >1 FPKM.

**Fig. 3:**
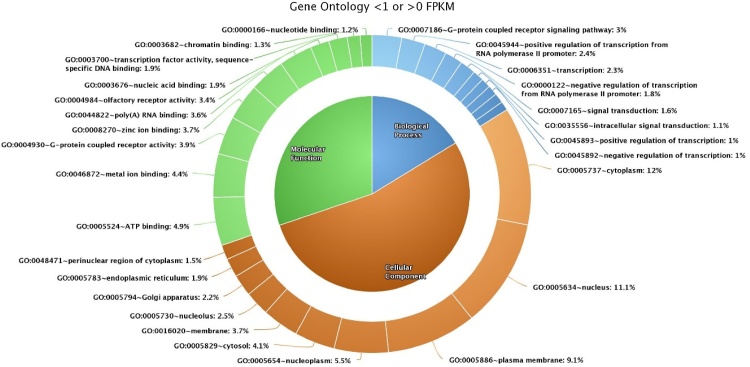
Top ten gene ontology (biological process, cellular component, molecular function) categories of transcripts >0 FPKM.

Gene ontology analysis was performed separately for transcripts with FPKM > 1 and FPKM > 0. Among the 431 transcripts (FPKM > 1) inculcated for gene ontology analysis, 241 were functionally annotated. Functional annotation by the DAVID software revealed 36 biological processes, 22 cellular components, 18 molecular functions (Suppl. Table 1) and five KEGG pathways (Table 4). Among the 13,383 transcripts with FPKM > 0, 1130 were functionally annotated, in which 131 biological processes, 74 cellular components, 60 molecular functions and 75 KEGG pathways were identified. The top ten categories of biological processes, cellular components and molecular functions of FPKM > 1 (Fig. 2) and FPKM > 0 (Fig. 3) were plotted in the form of a Donut pie chart and listed in Supplemental Table 2.

**Table 4:** KEGG pathway of transcripts >1 FPKM.

SI.NO	Term	Count	Percentage	*P* value
1	bta03010:Ribosome	47	19.50	1.83E-50
2	bta05012:Parkinson's disease	10	4.15	0.001006
3	bta00190:Oxidative phosphorylation	9	3.73	0.002402
4	bta03040:Spliceosome	6	2.49	0.065984
5	bta04666:Fc gamma R-mediated phagocytosis	5	2.07	0.054555

Among the GO categories of sperm transcripts having FPKM > 1, the top five biological processes include translation (16.2 %, 39 count, 1.14E-36), ribosomal small subunit assembly (2.9 %, seven count, 1.90E-08), cytoplasmic translation (2.9 %, seven count, 6.05E-07), nucleosome assembly (2.9 %, seven count, 0.00325) and nucleus organization (2.5 %, six count, 3.96E-07). Cellular components include nucleus (26.6 %, 64 count, 1.66E-05), extracellular exosome (24.5 %, 59 count, 5.95E-08), membrane (16.2 %, 39 count, 1.76E-10), cytosolic large ribosomal subunit (10.4 %, 25 count, 2.16E-29) and focal adhesion (10.4 %, 25 count, 5.81E-12). Molecular functions include structural constituent of ribosome (19.5 %, 47 count, 4.33E-43), poly (A) RNA binding (17.4 %, 42 count, 3.86E-13), DNA binding (5.8 %, 14 count, 0.096578), RNA binding (5.4 %, 13 count, 2.28E-04) and ubiquitin protein ligase binding (2.9 %, seven count, 0.004609). The predominant sperm genes involved in the biological processes (BP), cellular component (CC) and molecular functions (MF) are reported in Table 5.

**Table 5:** Predominant genes involved in BP, CC, and MF of sperm transcripts with FPKM > 1.

Gene Ontology	Genes
BP	
Translation	*FAU, RPL10, RPL11, RPL13, RPL13A, RPL14, RPL18, RPL23, RPL27A, RPL3, RPL30, RPL32, RPL34, RPL35, RPL36AL, RPL37, RPL37A, RPL38, RPL5, RPS11, RPS12, RPS14, RPS15, RPS17, RPS19, RPS2, RPS20, RPS23, RPS24, RPS27, RPS27A, RPS28, RPS3, RPS5, RPS7, RPS8, RPS9, SLC25A15*
MF	
Structural component of ribosome	*FAU, RPL10, RPL11, RPL13, RPL13A, RPL14, RPL18, RPL23, RPL26, RPL27A, RPL3, RPL30, RPL32, RPL34, RPL35, RPL35A, RPL36AL, RPL37, RPL37A, RPL38, RPL5, RPS8, RPS10, RPS11, RPS12, RPS14, RPS15, RPS17, RPS19, RPS2, RPS20, RPS23, RPS24, RPS27, RPS27A, RPS28, RPS3, RPS5, RPS7, RPS8, RPS9, RPLP0, RPLP1, RPLP2, SLC25A15, UBA52*
CC	
Nucleus	*DDX5, RTF1, THAP1, EEF1A1, RRP1B, RPL11, RPL18, RPL23, RPL3, RPL34, RPL5, RPL8, RPS10, RPS11, RPS19, RPS23, RPS25, RPS27A, RPS3, RPS7, RPS9, TNP2*

### 3.3 Pathway enrichment and network analysis of sperm transcripts

**Fig. 4:**
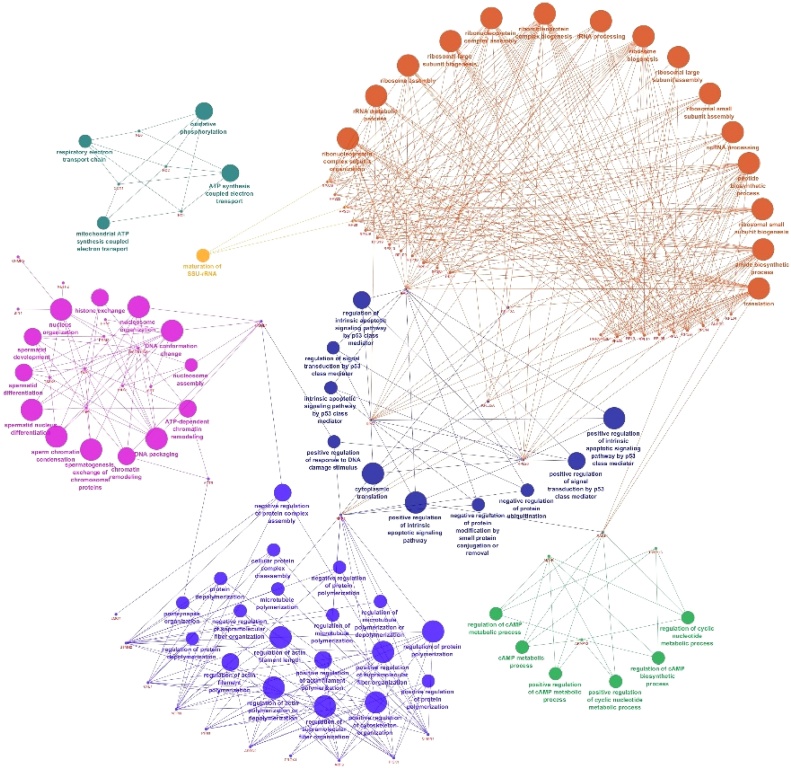
Network analysis of genes with biological process (BP) activity with FPKM > 1.

**Fig. 5:**
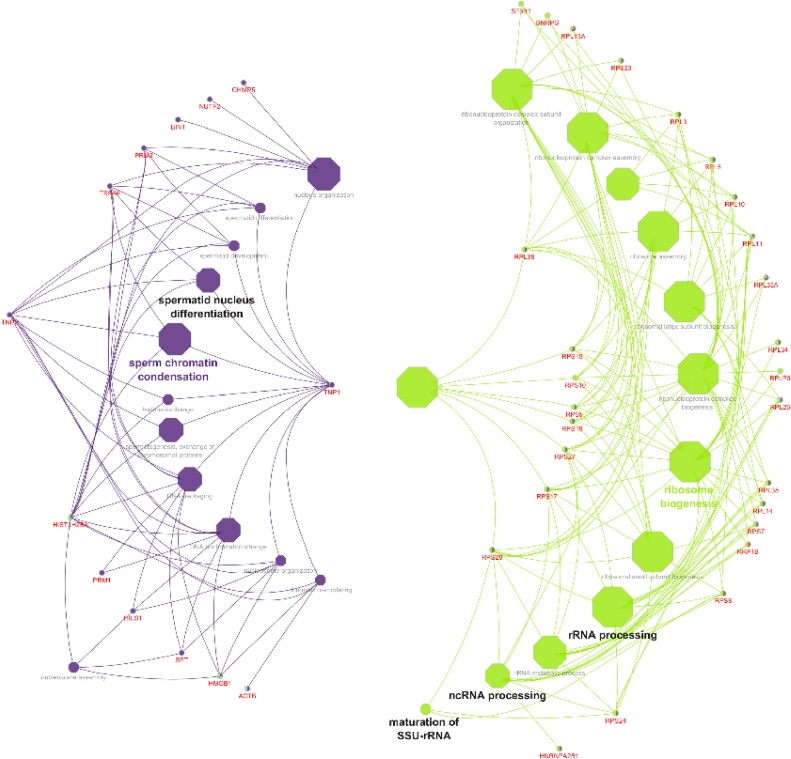
Network analysis of genes with selected biological process (BP) with FPKM > 1.

**Fig. 6:**
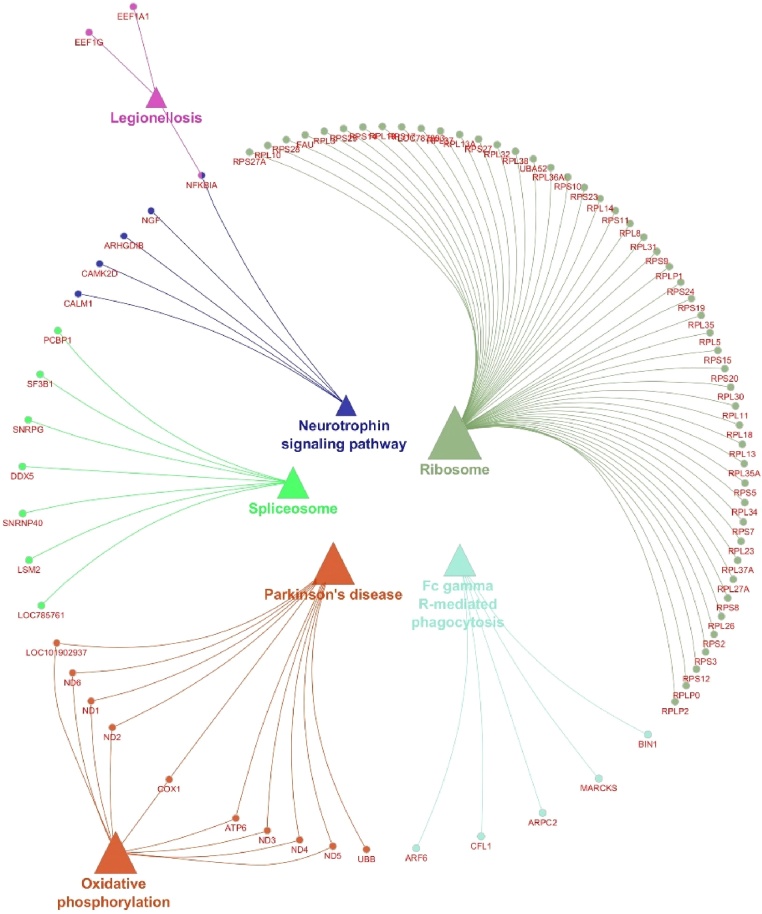
Network analysis of pathways and gene interaction with sperm transcripts FPKM > 1.

Pathway enrichment of genes specific to sperm transcripts with FPKM > 1 (Table 6) revealed involvement related to ribosome (Suppl. Fig. 1), Parkinson’s disease, oxidative phosphorylation (Suppl. Fig. 2) and spliceosome. Network and interaction analysis of genes with FPKM > 1 using ClueGo-Cytoscape revealed 150 BP (Fig. 4), 32 CC, 18 MF and seven pathways (Suppl. Fig. 3ab). Among these, selected BP (Fig. 5) (Suppl. Table 3), CC, MF and KEGG pathways (Fig. 6) and its interaction of genes specific to spermatogenesis, embryo development and fertilization are represented in Supplemental Table 4.

**Table 6:** Pathway enrichment of genes specific to sperm transcripts with FPKM > 1.

Pathway	Genes
Ribosome	*FAU, LOC85691, RPL10, RPL11, RPL13, RPL13A, RPL14, RPL18, RPL23, RPL26, RPL27A, RPL3, RPL30, RPL31, RPL32, RPL34, RPL35, RPL35A, RPL37, RPL37A, RPL38, RPL5, RPS8, RPS10, RPS11, RPS12, RPS14, RPS15, RPS17, RPS19, RPS2, RPS20, RPS23, RPS24, RPS25, RPS27, RPS27A, RPS28, RPS3, RPS5, RPS7, RPS8, RPS9, RPLP0, RPLP1, RPLP2, UBA52*
Parkinson disease	*COX1, ATP6, ND1, ND2, ND3, ND4, ND5, ND6, UBB, LOC101902937*
Oxidative phosphorylation	*ATP6, ND1, ND2, ND3, ND4, ND5, ND6, LOC101902937, COX1*
Spliceosome	*DDX5, LSM2, HNRNPA1, PCBP1, SNRNP40, SF3B1*

### 3.4 Real time mRNA transcript relative abundance analysis of selected genes

**Fig. 7:**
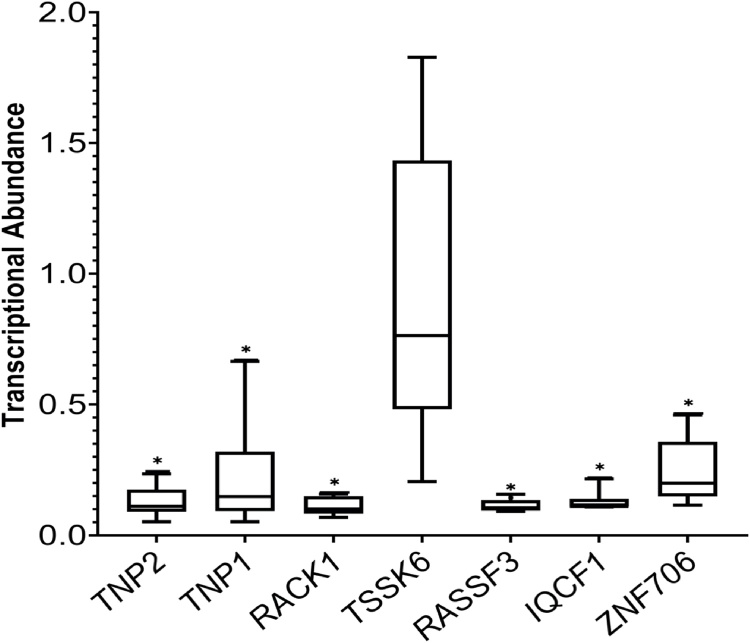
Box-plots indicating the individual variations in transcriptional abundance of selected genes (RT-qPCR analysis) in crossbred bull spermatozoa (n = 6 bulls).

Sperm transcriptional abundances of selected genes (*TNP2, TNP1, RACK1, TSSK6, RASSF3, IQCF1 and ZNF706*) are depicted in Fig. 7. There were considerable variations in the sperm transcriptional abundance among the bulls. Results with use of the Wilcoxon signed rank test indicated the relative abundances of *TNP2*, *TNP1*, *RACK1*, *RASSF3*, *IQCF1* transcripts and *ZNF706* genes was markedly different among bulls.

## 4 Discussion

Sperm RNA has a function in spermatogenesis, sperm maturation, sperm function, fertilization, oocyte genome activation, early embryogenesis and placental development (Selvaraju et al., 2018). The first reports on sperm transcriptome profiling were published for humans (Sendler et al., 2013), cattle (Card et al., 2013) and horses (Das et al., 2013). Previous studies of sperm transcriptomic profiling were performed on purebred and indigenous bulls to ascertain global transcriptomes or to study the transcripts based on varying fertility status. Developing a basic profile of sperm transcripts in crossbred bulls, however, is a pre-requisite to understand fertility-associated alterations. To the best of our knowledge, the present study is the first report on global transcriptomic profiling of crossbred bull spermatozoa.

The global transcriptomic profiling of cryopreserved crossbred bull spermatozoa has revealed transcripts for 13,814 genes. The number of sperm transcripts observed in the present study are consistent with values from earlier reports on bull sperm (Card et al., 2013; Selvaraju et al., 2017; Raval et al., 2019). In the present study, the large proportion of coding RNAs reported indicates there was efficacy of library preparation and sequencing. In the present study, the top ten abundant sperm transcripts were consisted of non-coding RNAs (rRNA, miRNA, misc_RNA). Several researchers have reported the presence of non-coding RNAs in livestock and human sperm (Sendler et al., 2013; Card et al., 2013; Das et al., 2013; Sellem et al., 2020), with functions in spermatogenesis, fertilization and embryogenesis (Zhang et al., 2017; Capra et al., 2017) as well as epigenetic modification (Jodar et al., 2013). Although the counts of non-coding RNAs were proportionately less than the protein coding RNAs, the relative abundances of mRNA transcripts was in a greater FPKM range. Consistent with this finding, Card et al. (2013) also reported non-coding RNAs among the top transcripts with the greatest FPKM. Similarly, a large proportion of non-coding RNAs in sperm transcriptomic profiling compared to protein coding were reported previously by Luk et al. (2014) and Raval et al. (2019). Wang et al. (2019), studied coding and non-coding RNAs in Holstein bull sperm and revealed that lncRNAs (TCONS_00041733) could regulate the relative abundance of mRNA transcript for protein-coding genes (*EFNA1*) and pathways by regulating sperm motility. Recently, Sellem et al. (2020) reported there were various small non-coding RNAs (sncRNAs) in different breeds of bulls with breed-specific patterns. After excluding non-coding RNAs, pseudogenes and processed pseudogenes, the top ten transcript (FPKM > 1) codes for protein include *PRM1, HMGB4, CCDC181, CHMP5, TNP2, RPS28, RPL37, TPT1, ENSBTAG00000047411* and *ANKRD9* genes. Furthermore, Real time gene expression analysis using RT-qPCR also confirmed the presence of of *TNP2*, *TNP1*, *RACK1*, *TSSK6*, *RASSF3*, *IQCF1* and *ZNF706* mRNA transcripts as validation of procedures used. The possible function of these transcripts is subsequently discussed in this manuscript.

Protamine 1 (*PRM1*) is a nuclear protein involved in cell differentiation, chromosome condensation, stabilizing sperm DNA and spermatogenesis (Oliva, 2006; Jodar and Oliva, 2014). It is the most abundant transcript present in bull sperm (Card et al., 2013, 2017; Selvaraju et al., 2017; Raval et al., 2019; Singh et al., 2019), and also in the present study based on results from these evaluations. High mobility group protein B4 (*HMGB4*) is the second most abundant mRNA transcript present in crossbred bull spermatozoa. The protein encoded by this gene is a potential transcriptional repressor with functional properties of chromatin binding (Catena et al., 2009; Card et al., 2013). Coiled-coil domain containing 181 (*CCDC181*) is a microtubule-binding protein that localizes to the microtubular manchette of elongating spermatids and is a candidate marker for disturbed ciliary motility condition in males (Schwarz et al., 2017). Charged multivesicular body protein 5 (*CHMP5*) has a function in male gamete generation, sperm function, and regulation of late endosome function during embryogenesis and spermatogenesis (Selvaraju et al., 2017; Card et al., 2017). Nuclear transition protein 2 (*TNP2*) transcript is associated with sub-optimal semen quality (Yathish et al., 2017), teratozoospermia (Savadi-shiraz et al., 2015) and asthenozoospermia (Jedrzejczak et al., 2007). The *TNP1* and *TNP2* genes are conserved, and the proteins encoded by these genes have important functions during the embryonic nuclear transition period. Ribosomal protein 28 (*RPS28*) and Ribosomal protein 37 (*RPL37*) transcripts were also observed in the bull sperm transcriptome (Card et al., 2017; Selvaraju et al., 2017; Raval et al., 2019). Translationally-controlled tumor protein (*TCTP*) is involved in the process of apoptosis and cellular differentiation, and controlling sperm functions (Arcuri et al., 2004) and this protein is in relatively lesser abundance in oligozoospermic patients (Montjean et al., 2012). Ankyrin repeat domain 9 (*ANKRD9*) is involved in protein localization to the plasma membrane and ion channel binding and is down regulated in asthenozoospermic patients (Jodar et al., 2012).

Some of the following sperm transcripts of protein coding genes having functional relevance to sperm formation, maturation, fertilization, and embryo development were detected in the present study with FPKM > 1. Tyrosine 3-monooxygenase activation protein zeta (*YWHAZ*) has a function in male gamete formation, sperm function, protein phosphorylation and oocyte meiosis (Selvaraju et al., 2017). Thymosin beta 10 (*TMSB10*) is involved in the process of actin filament organization and this gene is expressed in highly fertile bulls (Kropp et al., 2017). The spermatid nuclear transition protein 1 (*TNP1*) has a function in chromatin remodeling, flagellated sperm motility, spermatid nucleus elongation and spermatogenesis (Wu et al., 2009); this transcript was also observed by Ganguly et al. (2016) and Selvaraju et al. (2017) in bull sperm. Testis specific serine kinase 6 (*TSSK6*) has a function in intracellular signal transduction, peptidyl-serine phosphorylation, protein phosphorylation sperm chromatin condensation, sperm function and gamete fusion (Sosnik et al., 2009; Li et al., 2011; Selvaraju et al., 2018). Sperm mitochondria associated cysteine rich protein (*SMCP*) is involved in regulating sperm motility (Nayernia et al., 2002). Both *TSSK6* and *SMCP* were also detected in bull sperm by Card et al. (2013). The DnaJ homolog subfamily C member 2 (*DNAJC2*), as reported by Selvaraju et al. (2017) in bull sperm, is involved in stem cell differentiation and early embryonic development (Helary et al., 2019). In addition, *RACK1*, a multifaceted scaffolding protein interacts with the ribosomal components (Adams et al., 2011) and regulates protein phosphorylation. The IQ motif containing F1 (*IQCF1*) is an acrosomal protein localized mostly in the acrosome of spermatozoa and spermatids; it is associated with sperm capacitation and acrosome reaction, and is also involved in tyrosine phosphorylation (Fang et al., 2015). Zinc-containing metalloenzymes are associated with sperm functions (Kerns et al., 2018); the zinc finger protein *ZNF706* is important for male and female fertility.

Gene ontology analysis of transcripts with FPKM > 1 revealed that translation is the predominant biological process in bull sperm, which possess abundant transcripts of ribosomal proteins. In bull sperm, ribosomal RNAs are detected as either intact or degraded (Selvaraju et al., 2017) and could be packed during the process of spermatogenesis (Montjean et al., 2012). The primary function of ribosomal RNA is protein synthesis which is essential for maintaining the functions of mitochondria and sperm motility (Bansal et al., 2015). The ribosomal pathway is predominantly identified in crossbred bull spermatozoa followed by Parkinson’s disease, oxidative phosphorylation and spliceosome. The involvement of transcripts in the translation process and ribosomal pathway in the present study is not evident because the ribosome gradually disappears during chromatin compaction resulting in transcriptional and translational inactivity of mature sperm (Jodar and Oliva, 2014). Zhao et al., 2009 and Gur and Breitbart (2006), however, reported that unlike cytoplasmic ribosomes, mitochondrial ribosomes translate proteins during sperm capacitation until fertilization. Ribosomal proteins and the functions in mature sperm, therefore, need to be explored further. The connection between bull sperm transcripts and Parkinson’s disease is unknown. It may, however, indicate the relation between the paternal sperm transcripts and neuronal health of offspring, which was recently studied by Tomoiaga et al. (2020) using single cell RNA sequencing of sperm. Other pathways such as the oxidative phosphorylation, are primarily involved in ATP production as a source of energy for sperm motility (Sengupta et al., 2020), whereas spliceosomes are involved in spermatogonial proliferation and differentiation (Wu et al., 2016). Results from the present study on individual variations in transcriptional abundance in crossbred bull spermatozoa indicate a possibility of the association with sperm quality and fertilizing potential.

## 5 Conclusion

Collectively, results from global transcriptomic profiling of Holstein Friesian crossbred bull spermatozoa indicates that sperm transcripts were involved in functions such as the structural constituent of ribosome and translation, as well as pathways such as ribosome, oxidative phosphorylation and spliceosome. There may not be involvement of transcripts in the translation process and ribosomal pathway that was evaluated in the present study because the ribosome gradually disappears during chromatin compaction, resulting in the lack of translational capacity of mature sperm. Further study, however, is needed to confirm or refute translational inactivity in sperm. These preliminary findings expand the knowledge base on sperm transcripts and warrants further studies using larger sample sizes to understand the possible implications of transcriptomic variations on semen quality and fertility.

## CRediT authorship contribution statement

**Mani Arul Prakash:** Writing - original draft, Methodology, Data curation, Formal analysis. **Arumugam Kumaresan:** Conceptualization, Methodology, Project administration, Supervision, Writing - review & editing. **Manish Kumar Sinha:** Conceptualization, Methodology, Formal analysis. **Elango Kamaraj:** Writing - original draft. **Tushar Kumar Mohanty:** Methodology, Writing - review & editing. **Tirtha Kumar Datta:** Methodology, Writing - review & editing. **Jane M. Morrell:** Writing - review & editing.

## Declaration of Competing Interest

The authors declare that there is no conflict of interests
