# Supplementary material for: RNA-Seq analysis reveals functionally relevant coding and non-coding RNAs in crossbred bull spermatozoa
Source: Anim Reprod Sci. 2020 Nov;222:106621. doi: 10.1016/j.anireprosci.2020.106621 (PMC7607363; doi:10.1016/j.anireprosci.2020.106621)
Supplement: Supplementary file 1 [file mmc1.docx]

**
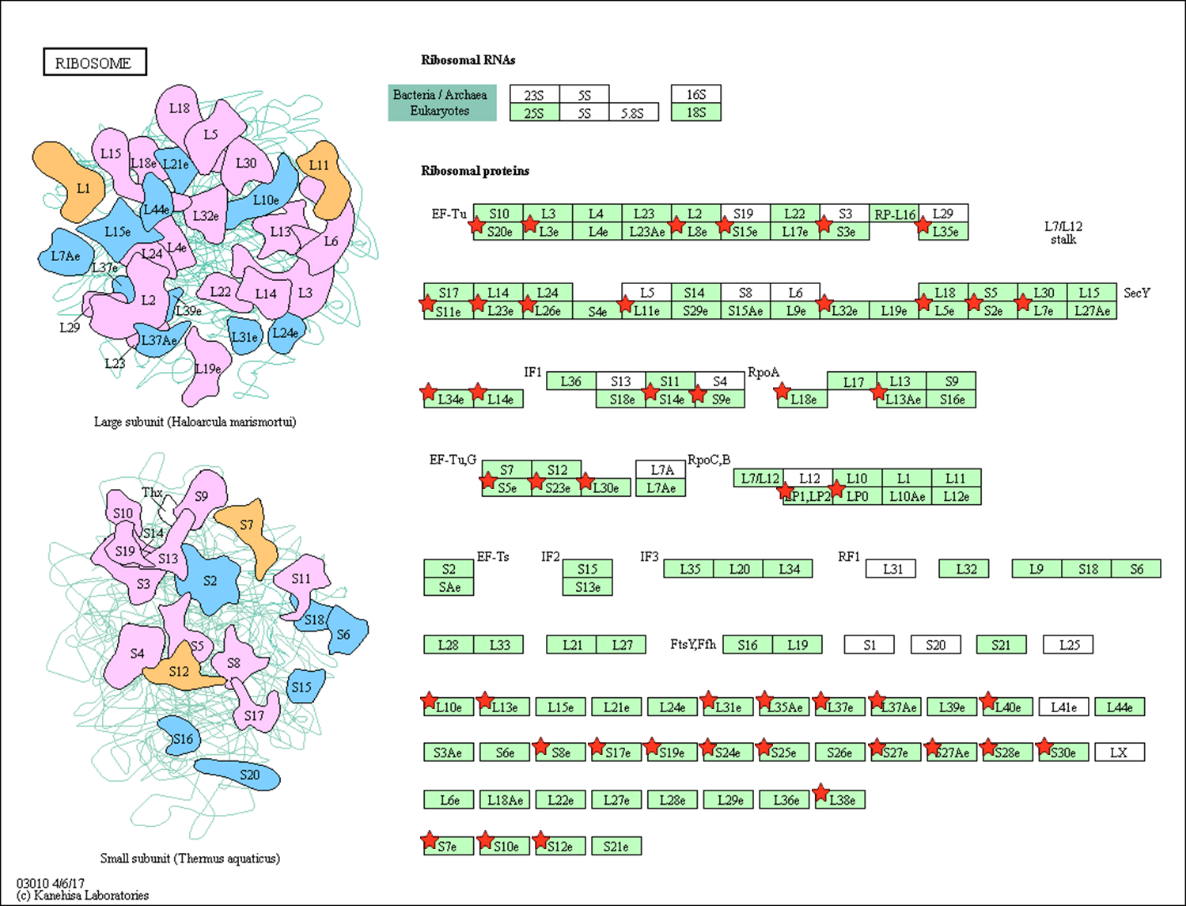
**

**Fig. S1: Spermatozoal transcripts with FPKM >1 involved in Ribosome pathway (KEGG)**

**
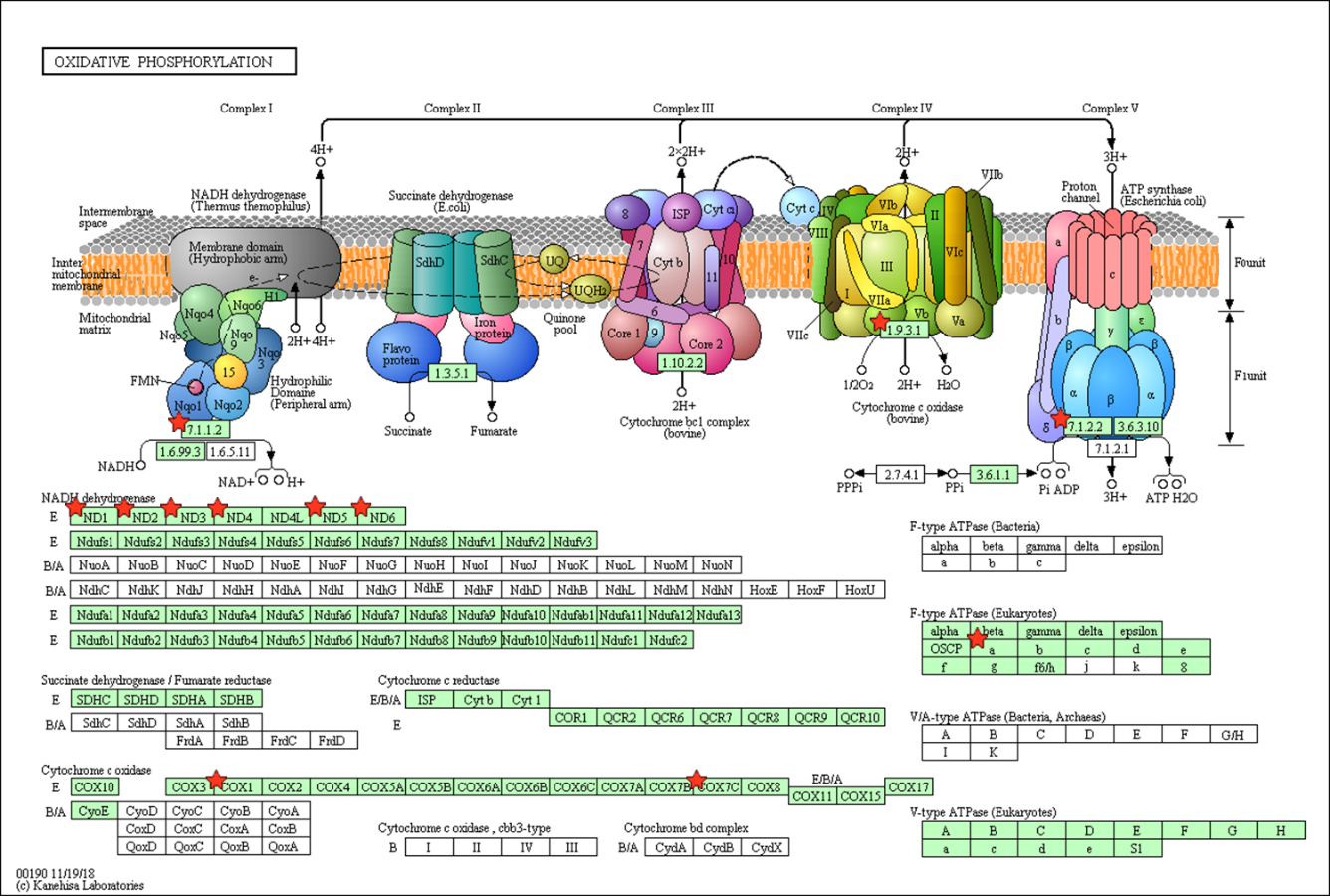
**

**Fig. S2: Spermatozoal transcripts with FPKM >1 involved in Oxidative Phosphorylation (KEGG)**

**
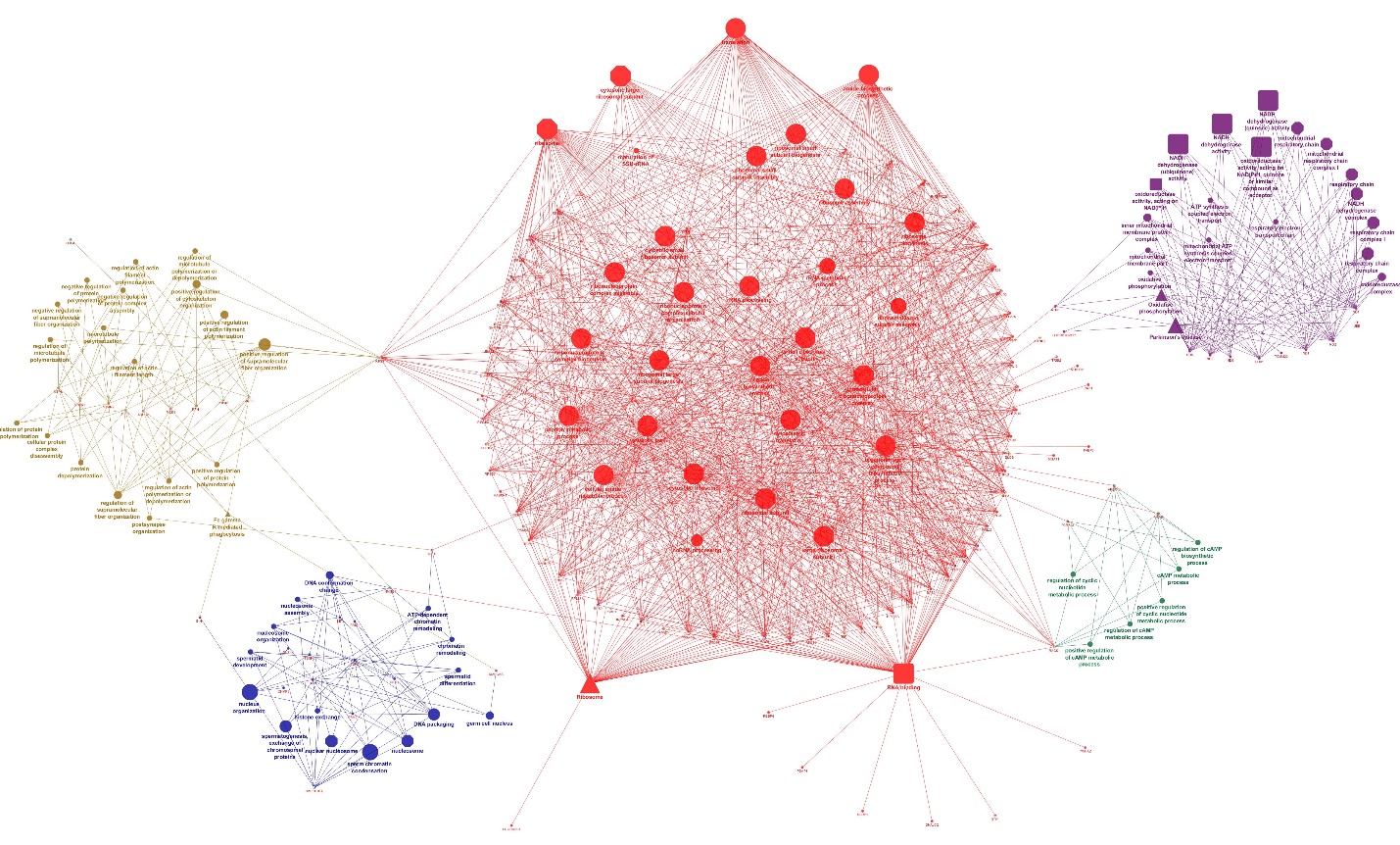
**

**Fig. S3a: Network analysis and interaction of genes of selected Gene Ontology components and pathway with FPKM >1**

**
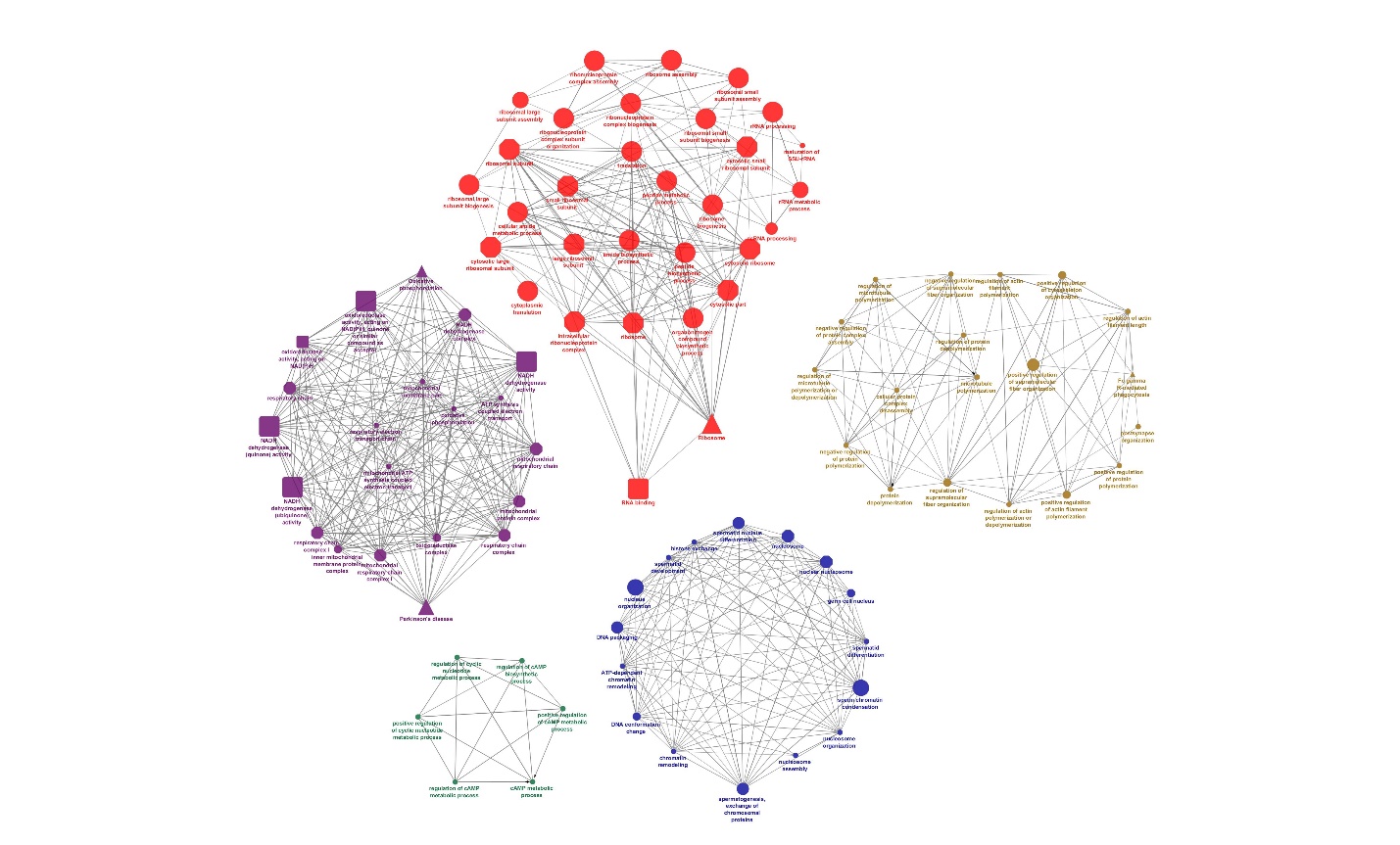
**

**Fig. S3b: Network analysis with selected Gene Ontology components and pathway with FPKM >1**
