# Supplementary material for: RNA-Seq analysis reveals functionally relevant coding and non-coding RNAs in crossbred bull spermatozoa
Source: Anim Reprod Sci. 2020 Nov;222:106621. doi: 10.1016/j.anireprosci.2020.106621 (PMC7607363; doi:10.1016/j.anireprosci.2020.106621)
Supplement: Supplementary file 2 [file mmc2.docx]

**Table S1: Gene ontology of transcripts >1 FPKM**

**Biological process**

| **SI.NO** | **Term** | **Count** | **Percentage** | **P value** |
| --- | --- | --- | --- | --- |
| 1 | GO:0006412~translation | 39 | 16.18 | 1.14E-36 |
| 2 | GO:0000028~ribosomal small subunit assembly | 7 | 2.90 | 1.90E-08 |
| 3 | GO:0002181~cytoplasmic translation | 7 | 2.90 | 6.05E-07 |
| 4 | GO:0006334~nucleosome assembly | 7 | 2.90 | 0.00325 |
| 5 | GO:0006997~nucleus organization | 6 | 2.49 | 3.96E-07 |
| 6 | GO:0006364~rRNA processing | 5 | 2.07 | 0.003079 |
| 7 | GO:0007286~spermatid development | 5 | 2.07 | 0.008772 |
| 8 | GO:0006414~translational elongation | 4 | 1.66 | 5.46E-04 |
| 9 | GO:0006605~protein targeting | 4 | 1.66 | 0.001039 |
| 10 | GO:0000027~ribosomal large subunit assembly | 4 | 1.66 | 0.002037 |
| 11 | GO:0048538~thymus development | 4 | 1.66 | 0.011831 |
| 12 | GO:0098609~cell-cell adhesion | 4 | 1.66 | 0.055261 |
| 13 | GO:0032496~response to lipopolysaccharide | 4 | 1.66 | 0.091619 |
| 14 | GO:0042692~muscle cell differentiation | 3 | 1.24 | 0.015506 |
| 15 | GO:0001895~retina homeostasis | 3 | 1.24 | 0.017573 |
| 16 | GO:0090200~positive regulation of release of cytochrome c from mitochondria | 3 | 1.24 | 0.024419 |
| 17 | GO:0042273~ribosomal large subunit biogenesis | 3 | 1.24 | 0.024419 |
| 18 | GO:0042254~ribosome biogenesis | 3 | 1.24 | 0.040747 |
| 19 | GO:0000462~maturation of SSU-rRNA from tricistronic rRNA transcript (SSU-rRNA, 5.8S rRNA, LSU-rRNA | 3 | 1.24 | 0.050077 |
| 20 | GO:0070527~platelet aggregation | 3 | 1.24 | 0.06009 |
| 21 | GO:1902340~negative regulation of chromosome condensation | 2 | 0.83 | 0.025587 |
| 22 | GO:0051170~nuclear import | 2 | 0.83 | 0.025587 |
| 23 | GO:0090230~regulation of centromere complex assembly | 2 | 0.83 | 0.025587 |
| 24 | GO:0031509~telomeric heterochromatin assembly | 2 | 0.83 | 0.025587 |
| 25 | GO:0045581~negative regulation of T cell differentiation | 2 | 0.83 | 0.038136 |
| 26 | GO:0060266~negative regulation of respiratory burst involved in inflammatory response | 2 | 0.83 | 0.038136 |
| 27 | GO:0030261~chromosome condensation | 2 | 0.83 | 0.050524 |
| 28 | GO:0003266~regulation of secondary heart field cardio blast proliferation | 2 | 0.83 | 0.050524 |
| 29 | GO:0031508~pericentric heterochromatin assembly | 2 | 0.83 | 0.050524 |
| 30 | GO:0051343~positive regulation of cyclic-nucleotide phosphodiesterase activity | 2 | 0.83 | 0.050524 |
| 31 | GO:0021707~cerebellar granule cell differentiation | 2 | 0.83 | 0.050524 |
| 32 | GO:0032464~positive regulation of protein homo-oligomerization | 2 | 0.83 | 0.074825 |
| 33 | GO:2000271~positive regulation of fibroblast apoptotic process | 2 | 0.83 | 0.074825 |
| 34 | GO:0051901~positive regulation of mitochondrial depolarization | 2 | 0.83 | 0.074825 |
| 35 | GO:0042773~ATP synthesis coupled electron transport | 2 | 0.83 | 0.074825 |
| 36 | GO:0003254~regulation of membrane depolarization | 2 | 0.83 | 0.086743 |
| 37 | GO:0010941~regulation of cell death | 2 | 0.83 | 0.098508 |

**Cellular Component**

| **SI.NO** | **Term** | **Count** | **Percentage** | **P value** |
| --- | --- | --- | --- | --- |
| 1 | GO:0005634~nucleus | 64 | 26.56 | 1.66E-05 |
| 2 | GO:0070062~extracellular exosome | 59 | 24.48 | 5.95E-08 |
| 3 | GO:0016020~membrane | 39 | 16.18 | 1.76E-10 |
| 4 | GO:0022625~cytosolic large ribosomal subunit | 25 | 10.37 | 2.16E-29 |
| 5 | GO:0005925~focal adhesion | 25 | 10.37 | 5.81E-12 |
| 6 | GO:0005654~nucleoplasm | 25 | 10.37 | 0.065683 |
| 7 | GO:0005730~nucleolus | 22 | 9.13 | 5.56E-05 |
| 8 | GO:0022627~cytosolic small ribosomal subunit | 19 | 7.88 | 6.51E-24 |
| 9 | GO:0005840~ribosome | 12 | 4.98 | 9.67E-10 |
| 10 | GO:0031012~extracellular matrix | 11 | 4.56 | 3.24E-06 |
| 11 | GO:0005743~mitochondrial inner membrane | 10 | 4.15 | 0.015945 |
| 12 | GO:0030529~intracellular ribonucleoprotein complex | 8 | 3.32 | 1.72E-05 |
| 13 | GO:0000786~nucleosome | 7 | 2.90 | 5.48E-04 |
| 14 | GO:0071013~catalytic step 2 spliceosome | 7 | 2.90 | 9.25E-04 |
| 15 | GO:0043005~neuron projection | 6 | 2.49 | 0.029533 |
| 16 | GO:0070469~respiratory chain | 5 | 2.07 | 3.65E-05 |
| 17 | GO:0005747~mitochondrial respiratory chain complex I | 5 | 2.07 | 0.003429 |
| 18 | GO:0030687~preribosome, large subunit precursor | 4 | 1.66 | 0.005314 |
| 19 | GO:0032587~ruffle membrane | 4 | 1.66 | 0.025669 |
| 20 | GO:0071011~precatalytic spliceosome | 3 | 1.24 | 0.049432 |
| 21 | GO:0001740~Barr body | 2 | 0.83 | 0.064569 |
| 22 | GO:0097433~dense body | 2 | 0.83 | 0.076976 |

**Molecular Function**

| **SI.NO** | **Term** | **Count** | **Percentage** | **P value** |
| --- | --- | --- | --- | --- |
| 1 | GO:0003735~structural constituent of ribosome | 47 | 19.50 | 4.33E-43 |
| 2 | GO:0044822~poly(A) RNA binding | 42 | 17.43 | 3.86E-13 |
| 3 | GO:0003677~DNA binding | 14 | 5.81 | 0.096578 |
| 4 | GO:0003723~RNA binding | 13 | 5.39 | 2.28E-04 |
| 5 | GO:0031625~ubiquitin protein ligase binding | 7 | 2.90 | 0.004609 |
| 6 | GO:0019843~rRNA binding | 6 | 2.49 | 6.56E-05 |
| 7 | GO:0008137~NADH dehydrogenase (ubiquinone activity) | 6 | 2.49 | 7.65E-05 |
| 8 | GO:0003729~mRNA binding | 6 | 2.49 | 0.012176 |
| 9 | GO:0008134~transcription factor binding | 5 | 2.07 | 0.018993 |
| 10 | GO:0019901~protein kinase binding | 5 | 2.07 | 0.023468 |
| 11 | GO:0070180~large ribosomal subunit rRNA binding | 4 | 1.66 | 2.42E-05 |
| 12 | GO:0098641~cadherin binding involved in cell-cell adhesion | 4 | 1.66 | 0.058487 |
| 13 | GO:0008017~microtubule binding | 4 | 1.66 | 0.07223 |
| 14 | GO:0015631~tubulin binding | 3 | 1.24 | 0.015018 |
| 15 | GO:0003746~translation elongation factor activity | 3 | 1.24 | 0.024371 |
| 16 | GO:0019904~protein domain specific binding | 3 | 1.24 | 0.098233 |
| 17 | GO:0097100~supercoiled DNA binding | 2 | 0.83 | 0.053279 |
| 18 | GO:0070063~RNA polymerase binding | 2 | 0.83 | 0.066152 |

**Table S2: TOP 10 Gene Ontology categories between FPKM >1 & FPKM >0**

|  | **>1 FPKM** |  |  |  | **FPKM >0** |  |  |
| --- | --- | --- | --- | --- | --- | --- | --- |
| **BP** |  | **Count** | **%** | **BP** |  | **Count** | **%** |
|  | GO:0006412~translation | 39 | 7.22 |  | GO:0007186~G-protein coupled receptor signaling pathway | 520 | 3.0 |
|  | GO:0000028~ribosomal small subunit assembly | 7 | 1.30 |  | GO:0045944~positive regulation of transcription from RNA polymerase II promoter | 416 | 2.4 |
|  | GO:0002181~cytoplasmic translation | 7 | 1.30 |  | GO:0006351~transcription, DNA-templated | 404 | 2.3 |
|  | GO:0006334~nucleosome assembly | 7 | 1.30 |  | GO:0000122~negative regulation of transcription from RNA polymerase II promoter | 302 | 1.8 |
|  | GO:0006997~nucleus organization | 6 | 1.11 |  | GO:0007165~signal transduction | 283 | 1.6 |
|  | GO:0006364~rRNA processing | 5 | 0.93 |  | GO:0035556~intracellular signal transduction | 194 | 1.1 |
|  | GO:0007286~spermatid development | 5 | 0.93 |  | GO:0045893~positive regulation of transcription, DNA-templated | 180 | 1.0 |
|  | GO:0006414~translational elongation | 4 | 0.74 |  | GO:0045892~negative regulation of transcription, DNA-templated | 174 | 1.0 |
|  | GO:0006605~protein targeting | 4 | 0.74 |  | GO:0006357~regulation of transcription from RNA polymerase II promoter | 171 | 1.0 |
|  | GO:0000027~ribosomal large subunit assembly | 4 | 0.74 |  | GO:0008285~negative regulation of cell proliferation | 154 | 0.9 |
|  | Total | 88 | 16.30 |  | Total | 2798 | 16.3 |
| **CC** |  | **Count** | **%** | **MF** |  | **Count** | **%** |
|  | GO:0005634~nucleus | 64 | 11.85 |  | GO:0005737~cytoplasm | 2069 | 12.0 |
|  | GO:0070062~extracellular exosome | 59 | 10.93 |  | GO:0005634~nucleus | 1912 | 11.1 |
|  | GO:0016020~membrane | 39 | 7.22 |  | GO:0005886~plasma membrane | 1566 | 9.1 |
|  | GO:0022625~cytosolic large ribosomal subunit | 25 | 4.63 |  | GO:0005654~nucleoplasm | 939 | 5.5 |
|  | GO:0005925~focal adhesion | 25 | 4.63 |  | GO:0005829~cytosol | 704 | 4.1 |
|  | GO:0005654~nucleoplasm | 25 | 4.63 |  | GO:0016020~membrane | 638 | 3.7 |
|  | GO:0005730~nucleolus | 22 | 4.07 |  | GO:0005730~nucleolus | 428 | 2.5 |
|  | GO:0022627~cytosolic small ribosomal subunit | 19 | 3.52 |  | GO:0005794~Golgi apparatus | 374 | 2.2 |
|  | GO:0005840~ribosome | 12 | 2.22 |  | GO:0005783~endoplasmic reticulum | 333 | 1.9 |
|  | GO:0031012~extracellular matrix | 11 | 2.04 |  | GO:0048471~perinuclear region of cytoplasm | 258 | 1.5 |
|  | Total | 301 | 55.74 |  | Total | 9221 | 53.6 |
| **MF** |  | **Count** | **%** | **MF** |  | **Count** | **%** |
|  | GO:0003735~structural constituent of ribosome | 47 | 8.70 |  | GO:0005524~ATP binding | 848 | 4.9 |
|  | GO:0044822~poly(A) RNA binding | 42 | 7.78 |  | GO:0046872~metal ion binding | 764 | 4.4 |
|  | GO:0003677~DNA binding | 14 | 2.59 |  | GO:0004930~G-protein coupled receptor activity | 668 | 3.9 |
|  | GO:0003723~RNA binding | 13 | 2.41 |  | GO:0008270~zinc ion binding | 636 | 3.7 |
|  | GO:0031625~ubiquitin protein ligase binding | 7 | 1.30 |  | GO:0044822~poly(A) RNA binding | 616 | 3.6 |
|  | GO:0019843~rRNA binding | 6 | 1.11 |  | GO:0004984~olfactory receptor activity | 577 | 3.4 |
|  | GO:0008137~NADH dehydrogenase (ubiquinone activity) | 6 | 1.11 |  | GO:0003676~nucleic acid binding | 326 | 1.9 |
|  | GO:0003729~mRNA binding | 6 | 1.11 |  | GO:0003700~transcription factor activity, sequence-specific DNA binding | 321 | 1.9 |
|  | GO:0008134~transcription factor binding | 5 | 0.93 |  | GO:0003682~chromatin binding | 229 | 1.3 |
|  | GO:0019901~protein kinase binding | 5 | 0.93 |  | GO:0000166~nucleotide binding | 211 | 1.2 |
|  | Total | 151 | 27.96 |  | Total | 5196 | 30.2 |
|  | Overall total | 540 | 100.00 |  | Overall Total | 17215 | 100.00 |

**Table S3: Selected Biological processes specific to spermatogenesis, embryo development and fertilization**

| **GoTerm** | **Term PValue** | **Term PValue Corrected with Bonferroni step down** | **Group PValue** | **Group PValue Corrected with Bonferroni step down** | **% Associated Genes** | **Nr. Genes** | **Associated Genes Found** |
| --- | --- | --- | --- | --- | --- | --- | --- |
| respiratory electron transport chain | 0.01 | 0.47 | 0.02 | 0.09 | 5.33 | 4.00 | *COX1, ND2, ND4, ND5* |
| oxidative phosphorylation | 0.01 | 0.45 | 0.02 | 0.09 | 5.56 | 4.00 | *COX1, ND2, ND4, ND5* |
| ATP synthesis coupled electron transport | 0.00 | 0.29 | 0.02 | 0.09 | 7.02 | 4.00 | *COX1, ND2, ND4, ND5* |
| mitochondrial ATP synthesis coupled electron transport | 0.03 | 0.51 | 0.02 | 0.09 | 5.66 | 3.00 | *COX1, ND2, ND4* |
| regulation of cyclic nucleotide metabolic process | 0.03 | 0.42 | 0.05 | 0.05 | 4.08 | 4.00 | *AKAP12, MC4R, P2RY13, RACK1* |
| positive regulation of cyclic nucleotide metabolic process | 0.03 | 0.37 | 0.05 | 0.05 | 5.08 | 3.00 | *AKAP12, MC4R, RACK1* |
| cAMP metabolic process | 0.03 | 0.48 | 0.05 | 0.05 | 4.17 | 4.00 | *AKAP12, MC4R, P2RY13, RACK1* |
| regulation of cAMP metabolic process | 0.01 | 0.46 | 0.05 | 0.05 | 5.13 | 4.00 | *AKAP12, MC4R, P2RY13, RACK1* |
| positive regulation of cAMP metabolic process | 0.02 | 0.47 | 0.05 | 0.05 | 6.67 | 3.00 | *AKAP12, MC4R, RACK1* |
| regulation of cAMP biosynthetic process | 0.04 | 0.31 | 0.05 | 0.05 | 4.55 | 3.00 | *AKAP12, MC4R, P2RY13* |
| nucleus organization | 0.00 | 0.00 | 0.00 | 0.03 | 8.00 | 8.00 | *BIN1, CHMP5, HIST1H2BA, NUTF2, PRM2, TNP1, TNP2, TSSK6* |
| chromatin remodeling | 0.01 | 0.45 | 0.00 | 0.03 | 4.42 | 5.00 | *ACTB, HIST1H2BA, HMGB1, TNP1, TNP2* |
| nucleosome organization | 0.00 | 0.23 | 0.00 | 0.03 | 4.92 | 6.00 | *HILS1, HIST1H2BA, HMGB1, SET, TNP1, TNP2* |
| DNA conformation change | 0.00 | 0.05 | 0.00 | 0.03 | 4.55 | 9.00 | *HILS1, HIST1H2BA, HMGB1, PRM1, PRM2, SET, TNP1, TNP2, TSSK6* |
| spermatid differentiation | 0.02 | 0.46 | 0.00 | 0.03 | 4.10 | 5.00 | *HIST1H2BA, PRM2, TNP1, TNP2, TSSK6* |
| DNA packaging | 0.00 | 0.01 | 0.00 | 0.03 | 6.16 | 9.00 | *HILS1, HIST1H2BA, HMGB1, PRM1, PRM2, SET, TNP1, TNP2, TSSK6* |
| ATP-dependent chromatin remodeling | 0.00 | 0.09 | 0.00 | 0.03 | 10.00 | 4.00 | *ACTB, HIST1H2BA, TNP1, TNP2* |
| nucleosome assembly | 0.03 | 0.42 | 0.00 | 0.03 | 4.08 | 4.00 | *HILS1, HIST1H2BA, HMGB1, SET* |
| spermatid development | 0.01 | 0.47 | 0.00 | 0.03 | 4.20 | 5.00 | *HIST1H2BA, PRM2, TNP1, TNP2, TSSK6* |
| spermatid nucleus differentiation | 0.00 | 0.01 | 0.00 | 0.03 | 20.00 | 4.00 | *HIST1H2BA, TNP1, TNP2, TSSK6* |
| histone exchange | 0.00 | 0.12 | 0.00 | 0.03 | 15.00 | 3.00 | *HIST1H2BA, TNP1, TNP2* |
| sperm chromatin condensation | 0.00 | 0.00 | 0.00 | 0.03 | 36.36 | 4.00 | *HIST1H2BA, TNP1, TNP2, TSSK6* |
| spermatogenesis, exchange of chromosomal proteins | 0.00 | 0.01 | 0.00 | 0.03 | 33.33 | 3.00 | *HIST1H2BA, TNP1, TNP2* |
| ribonucleoprotein complex biogenesis | 0.00 | 0.00 | 0.00 | 0.00 | 7.30 | 26.00 | *RPL10, RPL11, RPL13A, RPL14, RPL26, RPL3, RPL34, RPL35, RPL35A, RPL38, RPL5, RPLP0, RPS10, RPS15, RPS17, RPS19, RPS23, RPS24, RPS27, RPS28, RPS5, RPS7, RPS8, RRP1B, SF3B1, SNRPG* |
| ribosome biogenesis | 0.00 | 0.00 | 0.00 | 0.00 | 9.57 | 22.00 | *RPL10, RPL11, RPL14, RPL26, RPL3, RPL34, RPL35, RPL35A, RPL38, RPL5, RPLP0, RPS10, RPS15, RPS17, RPS19, RPS24, RPS27, RPS28, RPS5, RPS7, RPS8, RRP1B* |
| ribonucleoprotein complex subunit organization | 0.00 | 0.00 | 0.00 | 0.00 | 8.25 | 16.00 | *RPL10, RPL11, RPL13A, RPL3, RPL38, RPL5, RPS10, RPS15, RPS17, RPS19, RPS23, RPS27, RPS28, RPS5, SF3B1, SNRPG* |
| ribosomal large subunit biogenesis | 0.00 | 0.00 | 0.00 | 0.00 | 13.43 | 9.00 | *RPL10, RPL11, RPL14, RPL26, RPL3, RPL35, RPL35A, RPL38, RPL5* |
| ribosomal small subunit biogenesis | 0.00 | 0.00 | 0.00 | 0.00 | 16.92 | 11.00 | *RPL38, RPS10, RPS15, RPS17, RPS19, RPS24, RPS27, RPS28, RPS5, RPS7, RPS8* |
| ribonucleoprotein complex assembly | 0.00 | 0.00 | 0.00 | 0.00 | 8.65 | 16.00 | *RPL10, RPL11, RPL13A, RPL3, RPL38, RPL5, RPS10, RPS15, RPS17, RPS19, RPS23, RPS27, RPS28, RPS5, SF3B1, SNRPG* |
| ribosome assembly | 0.00 | 0.00 | 0.00 | 0.00 | 22.22 | 12.00 | *RPL10, RPL11, RPL3, RPL38, RPL5, RPS10, RPS15, RPS17, RPS19, RPS27, RPS28, RPS5* |
| ribosomal large subunit assembly | 0.00 | 0.00 | 0.00 | 0.00 | 17.24 | 5.00 | *RPL10, RPL11, RPL3, RPL38, RPL5* |
| ribosomal small subunit assembly | 0.00 | 0.00 | 0.00 | 0.00 | 50.00 | 8.00 | *RPL38, RPS10, RPS15, RPS17, RPS19, RPS27, RPS28, RPS5* |
| rRNA metabolic process | 0.00 | 0.00 | 0.00 | 0.00 | 6.29 | 11.00 | *RPL11, RPL14, RPL26, RPL35, RPS17, RPS24, RPS27, RPS28, RPS7, RPS8, RRP1B* |
| ncRNA processing | 0.00 | 0.01 | 0.00 | 0.00 | 4.36 | 12.00 | *HNRNPA2B1, RPL11, RPL14, RPL26, RPL35, RPS17, RPS24, RPS27, RPS28, RPS7, RPS8, RRP1B* |
| rRNA processing | 0.00 | 0.00 | 0.00 | 0.00 | 7.28 | 11.00 | *RPL11, RPL14, RPL26, RPL35, RPS17, RPS24, RPS27, RPS28, RPS7, RPS8, RRP1B* |
| maturation of SSU-rRNA | 0.02 | 0.49 | 0.00 | 0.00 | 6.38 | 3.00 | *RPS24, RPS28, RPS8* |
| postsynapse organization | 0.04 | 0.35 | 0.00 | 0.01 | 5.00 | 3.00 | *ACTB, CFL1, PICK1* |
| regulation of supramolecular fiber organization | 0.00 | 0.06 | 0.00 | 0.01 | 4.05 | 10.00 | *ARF6, ARPC2, CFL1, MTPN, PFN1, PICK1, PROX1, RPS3, STMN1, STMN2* |
| negative regulation of supramolecular fiber organization | 0.03 | 0.47 | 0.00 | 0.01 | 4.12 | 4.00 | *MTPN, PICK1, STMN1, STMN2* |
| positive regulation of supramolecular fiber organization | 0.00 | 0.03 | 0.00 | 0.01 | 5.59 | 8.00 | *ARF6, ARPC2, CFL1, PFN1, PICK1, PROX1, RPS3, STMN2* |
| negative regulation of protein complex assembly | 0.01 | 0.30 | 0.00 | 0.01 | 5.38 | 5.00 | *HMGB1, LMO4, MTPN, STMN1, STMN2* |
| regulation of actin filament length | 0.01 | 0.31 | 0.00 | 0.01 | 4.55 | 6.00 | *ARF6, ARPC2, CFL1, MTPN, PFN1, PICK1* |
| regulation of protein polymerization | 0.00 | 0.04 | 0.00 | 0.01 | 5.33 | 8.00 | *ARF6, ARPC2, MTPN, PFN1, PICK1, RPS3, STMN1, STMN2* |
| cellular protein complex disassembly | 0.03 | 0.52 | 0.00 | 0.01 | 4.21 | 4.00 | *CFL1, MTPN, STMN1, STMN2* |
| microtubule polymerization | 0.03 | 0.44 | 0.00 | 0.01 | 5.36 | 3.00 | *RPS3, STMN1, STMN2* |
| positive regulation of cytoskeleton organization | 0.00 | 0.05 | 0.00 | 0.01 | 5.06 | 8.00 | *ARF6, ARPC2, CFL1, PFN1, PICK1, PROX1, RPS3, STMN2* |
| regulation of microtubule polymerization or depolymerization | 0.03 | 0.44 | 0.00 | 0.01 | 5.36 | 3.00 | *RPS3, STMN1, STMN2* |
| positive regulation of protein polymerization | 0.02 | 0.54 | 0.00 | 0.01 | 4.35 | 4.00 | *ARF6, ARPC2, PICK1, RPS3* |
| regulation of protein depolymerization | 0.04 | 0.35 | 0.00 | 0.01 | 5.00 | 3.00 | *CFL1, MTPN, STMN2* |
| regulation of actin polymerization or depolymerization | 0.01 | 0.31 | 0.00 | 0.01 | 4.55 | 6.00 | *ARF6, ARPC2, CFL1, MTPN, PFN1, PICK1* |
| regulation of microtubule polymerization | 0.01 | 0.35 | 0.00 | 0.01 | 9.09 | 3.00 | *RPS3, STMN1, STMN2* |
| negative regulation of protein polymerization | 0.02 | 0.53 | 0.00 | 0.01 | 6.00 | 3.00 | *MTPN, STMN1, STMN2* |
| protein depolymerization | 0.02 | 0.47 | 0.00 | 0.01 | 5.00 | 4.00 | *CFL1, MTPN, STMN1, STMN2* |
| regulation of actin filament polymerization | 0.01 | 0.47 | 0.00 | 0.01 | 4.31 | 5.00 | *ARF6, ARPC2, MTPN, PFN1, PICK1* |
| positive regulation of actin filament polymerization | 0.06 | 0.06 | 0.00 | 0.01 | 4.11 | 3.00 | *ARF6, ARPC2, PICK1* |

**Table S4:** Interaction of genes specific to spermatogenesis, embryo development and fertilization of sperm transcripts with FPKM >1

| GOID | GOTerm | Ontology Source | Term PValue | Term PValue Corrected with Bonferroni step down | Group PValue | Group PValue Corrected with Bonferroni step down | GOLevels | GOGroups | % Associated Genes | Nr. Genes | Associated Genes Found |
| --- | --- | --- | --- | --- | --- | --- | --- | --- | --- | --- | --- |
| GO:0019079 | viral genome replication | GO_BiologicalProcess-_11.01.2018_00h00 | 0.05 | 0.20 | 0.05 | 0.10 | [5, 6] | Group02 | 4.35 | 3.00 | [PCBP1, PPIA, PROX1] |
| GO:0045598 | regulation of fat cell differentiation | GO_BiologicalProcess-_11.01.2018_00h00 | 0.01 | 0.36 | 0.01 | 0.07 | [4, 5] | Group04 | 5.21 | 5.00 | [ADIG, DUSP10, HES1, JDP2, TRIB2] |
| GO:0050805 | negative regulation of synaptic transmission | GO_BiologicalProcess-_11.01.2018_00h00 | 0.01 | 0.50 | 0.01 | 0.13 | [3, 4, 5, 6, 7, 8, 9] | Group06 | 7.14 | 3.00 | [CBLN1, PICK1, PNKD] |
| GO:0035821 | modification of morphology or physiology of other organism | GO_BiologicalProcess-_11.01.2018_00h00 | 0.02 | 0.58 | 0.02 | 0.16 | [3] | Group10 | 4.40 | 4.00 | [BCL2L11, CFL1, RPL30, RRP1B] |
| GO:0051817 | modification of morphology or physiology of other organism involved in symbiotic interaction | GO_BiologicalProcess-_11.01.2018_00h00 | 0.05 | 0.16 | 0.02 | 0.16 | [4] | Group10 | 4.23 | 3.00 | [BCL2L11, CFL1, RRP1B] |
| GO:0048524 | positive regulation of viral process | GO_BiologicalProcess-_11.01.2018_00h00 | 0.05 | 0.28 | 0.02 | 0.16 | [3, 4, 5, 6] | Group10 | 4.48 | 3.00 | [CD74, CFL1, RRP1B] |
| GO:0006414 | translational elongation | GO_BiologicalProcess-_11.01.2018_00h00 | 0.00 | 0.00 | 0.00 | 0.00 | [5, 6, 7, 8] | Group11 | 17.65 | 6.00 | [EEF1A1, EEF1G, ELOB, RACK1, RPLP1, RPLP2] |
| GO:2001022 | positive regulation of response to DNA damage stimulus | GO_BiologicalProcess-_11.01.2018_00h00 | 0.01 | 0.41 | 0.00 | 0.00 | [3, 4, 5, 6, 7] | Group12 | 5.97 | 4.00 | [DDX5, HMGB1, RPL26, RPS3] |
| GO:0030799 | regulation of cyclic nucleotide metabolic process | GO_BiologicalProcess-_11.01.2018_00h00 | 0.03 | 0.43 | 0.05 | 0.05 | [6, 7, 8, 9] | Group14 | 4.08 | 4.00 | [AKAP12, MC4R, P2RY13, RACK1] |
| GO:0030801 | positive regulation of cyclic nucleotide metabolic process | GO_BiologicalProcess-_11.01.2018_00h00 | 0.03 | 0.38 | 0.05 | 0.05 | [6, 7, 8, 9, 10] | Group14 | 5.08 | 3.00 | [AKAP12, MC4R, RACK1] |
| GO:0046058 | cAMP metabolic process | GO_BiologicalProcess-_11.01.2018_00h00 | 0.03 | 0.49 | 0.05 | 0.05 | [7, 8, 9, 10] | Group14 | 4.17 | 4.00 | [AKAP12, MC4R, P2RY13, RACK1] |
| GO:0030814 | regulation of cAMP metabolic process | GO_BiologicalProcess-_11.01.2018_00h00 | 0.01 | 0.51 | 0.05 | 0.05 | [7, 8, 9, 10, 11] | Group14 | 5.13 | 4.00 | [AKAP12, MC4R, P2RY13, RACK1] |
| GO:0030816 | positive regulation of cAMP metabolic process | GO_BiologicalProcess-_11.01.2018_00h00 | 0.02 | 0.51 | 0.05 | 0.05 | [7, 8, 9, 10, 11, 12] | Group14 | 6.67 | 3.00 | [AKAP12, MC4R, RACK1] |
| GO:0030817 | regulation of cAMP biosynthetic process | GO_BiologicalProcess-_11.01.2018_00h00 | 0.05 | 0.32 | 0.05 | 0.05 | [7, 8, 9, 10, 11, 12] | Group14 | 4.55 | 3.00 | [AKAP12, MC4R, P2RY13] |
| GO:0030010 | establishment of cell polarity | GO_BiologicalProcess-_11.01.2018_00h00 | 0.01 | 0.36 | 0.01 | 0.07 | [3] | Group15 | 5.10 | 5.00 | [ARF6, CFL1, HES1, RACK1, SPRY2] |
| GO:0048864 | stem cell development | GO_BiologicalProcess-_11.01.2018_00h00 | 0.03 | 0.39 | 0.01 | 0.07 | [4, 5] | Group15 | 5.17 | 3.00 | [CFL1, HAND2, HES1] |
| GO:0061008 | hepaticobiliary system development | GO_BiologicalProcess-_11.01.2018_00h00 | 0.01 | 0.38 | 0.01 | 0.07 | [4, 5] | Group15 | 6.25 | 4.00 | [ARF6, HES1, PROX1, TAF10] |
| GO:0001889 | liver development | GO_BiologicalProcess-_11.01.2018_00h00 | 0.01 | 0.36 | 0.01 | 0.07 | [5, 6, 7] | Group15 | 6.56 | 4.00 | [ARF6, HES1, PROX1, TAF10] |
| GO:0014031 | mesenchymal cell development | GO_BiologicalProcess-_11.01.2018_00h00 | 0.03 | 0.38 | 0.01 | 0.07 | [4, 5, 6, 7, 8] | Group15 | 5.08 | 3.00 | [CFL1, HAND2, HES1] |
| GO:0014033 | neural crest cell differentiation | GO_BiologicalProcess-_11.01.2018_00h00 | 0.04 | 0.35 | 0.01 | 0.07 | [5, 6, 7, 8] | Group15 | 4.84 | 3.00 | [CFL1, HAND2, HES1] |
| GO:0014032 | neural crest cell development | GO_BiologicalProcess-_11.01.2018_00h00 | 0.03 | 0.51 | 0.01 | 0.07 | [5, 6, 7, 8, 9] | Group15 | 5.45 | 3.00 | [CFL1, HAND2, HES1] |
| GO:0043500 | muscle adaptation | GO_BiologicalProcess-_11.01.2018_00h00 | 0.03 | 0.51 | 0.03 | 0.16 | [2, 4] | Group16 | 5.45 | 3.00 | [CAMK2D, HAND2, MTPN] |
| GO:0014896 | muscle hypertrophy | GO_BiologicalProcess-_11.01.2018_00h00 | 0.01 | 0.49 | 0.03 | 0.16 | [4] | Group16 | 7.50 | 3.00 | [CAMK2D, HAND2, MTPN] |
| GO:0043502 | regulation of muscle adaptation | GO_BiologicalProcess-_11.01.2018_00h00 | 0.01 | 0.50 | 0.03 | 0.16 | [3, 4, 5, 6] | Group16 | 7.32 | 3.00 | [CAMK2D, HAND2, MTPN] |
| GO:0014742 | positive regulation of muscle hypertrophy | GO_BiologicalProcess-_11.01.2018_00h00 | 0.00 | 0.06 | 0.03 | 0.16 | [3, 4, 5, 6, 7] | Group16 | 20.00 | 3.00 | [CAMK2D, HAND2, MTPN] |
| GO:0014897 | striated muscle hypertrophy | GO_BiologicalProcess-_11.01.2018_00h00 | 0.01 | 0.49 | 0.03 | 0.16 | [5] | Group16 | 7.50 | 3.00 | [CAMK2D, HAND2, MTPN] |
| GO:0010611 | regulation of cardiac muscle hypertrophy | GO_BiologicalProcess-_11.01.2018_00h00 | 0.00 | 0.24 | 0.03 | 0.16 | [4, 5, 6, 7] | Group16 | 12.00 | 3.00 | [CAMK2D, HAND2, MTPN] |
| GO:0014743 | regulation of muscle hypertrophy | GO_BiologicalProcess-_11.01.2018_00h00 | 0.00 | 0.25 | 0.03 | 0.16 | [5, 6] | Group16 | 11.54 | 3.00 | [CAMK2D, HAND2, MTPN] |
| GO:0003300 | cardiac muscle hypertrophy | GO_BiologicalProcess-_11.01.2018_00h00 | 0.01 | 0.48 | 0.03 | 0.16 | [6] | Group16 | 7.89 | 3.00 | [CAMK2D, HAND2, MTPN] |
| GO:0010613 | positive regulation of cardiac muscle hypertrophy | GO_BiologicalProcess-_11.01.2018_00h00 | 0.00 | 0.06 | 0.03 | 0.16 | [4, 5, 6, 7, 8] | Group16 | 20.00 | 3.00 | [CAMK2D, HAND2, MTPN] |
| GO:0006997 | nucleus organization | GO_BiologicalProcess-_11.01.2018_00h00 | 0.00 | 0.00 | 0.00 | 0.03 | [4] | Group17 | 8.00 | 8.00 | [BIN1, CHMP5, HIST1H2BA, NUTF2, PRM2, TNP1, TNP2, TSSK6] |
| GO:0006338 | chromatin remodeling | GO_BiologicalProcess-_11.01.2018_00h00 | 0.01 | 0.49 | 0.00 | 0.03 | [4, 6] | Group17 | 4.42 | 5.00 | [ACTB, HIST1H2BA, HMGB1, TNP1, TNP2] |
| GO:0034728 | nucleosome organization | GO_BiologicalProcess-_11.01.2018_00h00 | 0.00 | 0.25 | 0.00 | 0.03 | [4, 5, 6] | Group17 | 4.92 | 6.00 | [HILS1, HIST1H2BA, HMGB1, SET, TNP1, TNP2] |
| GO:0071103 | DNA conformation change | GO_BiologicalProcess-_11.01.2018_00h00 | 0.00 | 0.06 | 0.00 | 0.03 | [5] | Group17 | 4.55 | 9.00 | [HILS1, HIST1H2BA, HMGB1, PRM1, PRM2, SET, TNP1, TNP2, TSSK6] |
| GO:0048515 | spermatid differentiation | GO_BiologicalProcess-_11.01.2018_00h00 | 0.02 | 0.50 | 0.00 | 0.03 | [3, 4, 6, 7, 8] | Group17 | 4.10 | 5.00 | [HIST1H2BA, PRM2, TNP1, TNP2, TSSK6] |
| GO:0006323 | DNA packaging | GO_BiologicalProcess-_11.01.2018_00h00 | 0.00 | 0.01 | 0.00 | 0.03 | [6] | Group17 | 6.16 | 9.00 | [HILS1, HIST1H2BA, HMGB1, PRM1, PRM2, SET, TNP1, TNP2, TSSK6] |
| GO:0043044 | ATP-dependent chromatin remodeling | GO_BiologicalProcess-_11.01.2018_00h00 | 0.00 | 0.11 | 0.00 | 0.03 | [5, 7] | Group17 | 10.00 | 4.00 | [ACTB, HIST1H2BA, TNP1, TNP2] |
| GO:0006334 | nucleosome assembly | GO_BiologicalProcess-_11.01.2018_00h00 | 0.03 | 0.43 | 0.00 | 0.03 | [5, 6, 7, 8] | Group17 | 4.08 | 4.00 | [HILS1, HIST1H2BA, HMGB1, SET] |
| GO:0007286 | spermatid development | GO_BiologicalProcess-_11.01.2018_00h00 | 0.01 | 0.50 | 0.00 | 0.03 | [4, 5, 6, 7, 8, 9] | Group17 | 4.20 | 5.00 | [HIST1H2BA, PRM2, TNP1, TNP2, TSSK6] |
| GO:0007289 | spermatid nucleus differentiation | GO_BiologicalProcess-_11.01.2018_00h00 | 0.00 | 0.01 | 0.00 | 0.03 | [3, 4, 5, 6, 7, 8, 9, 10] | Group17 | 20.00 | 4.00 | [HIST1H2BA, TNP1, TNP2, TSSK6] |
| GO:0043486 | histone exchange | GO_BiologicalProcess-_11.01.2018_00h00 | 0.00 | 0.13 | 0.00 | 0.03 | [5, 6, 7, 8] | Group17 | 15.00 | 3.00 | [HIST1H2BA, TNP1, TNP2] |
| GO:0035092 | sperm chromatin condensation | GO_BiologicalProcess-_11.01.2018_00h00 | 0.00 | 0.00 | 0.00 | 0.03 | [3, 4, 5, 6, 7, 8, 9, 10, 11] | Group17 | 36.36 | 4.00 | [HIST1H2BA, TNP1, TNP2, TSSK6] |
| GO:0035093 | spermatogenesis, exchange of chromosomal proteins | GO_BiologicalProcess-_11.01.2018_00h00 | 0.00 | 0.01 | 0.00 | 0.03 | [3, 4, 5, 6, 7, 8, 9, 10, 11, 12] | Group17 | 33.33 | 3.00 | [HIST1H2BA, TNP1, TNP2] |
| GO:0099173 | postsynapse organization | GO_BiologicalProcess-_11.01.2018_00h00 | 0.04 | 0.36 | 0.00 | 0.01 | [3, 4] | Group18 | 5.00 | 3.00 | [ACTB, CFL1, PICK1] |
| GO:1902903 | regulation of supramolecular fiber organization | GO_BiologicalProcess-_11.01.2018_00h00 | 0.00 | 0.07 | 0.00 | 0.01 | [4, 5] | Group18 | 4.05 | 10.00 | [ARF6, ARPC2, CFL1, MTPN, PFN1, PICK1, PROX1, RPS3, STMN1, STMN2] |
| GO:1902904 | negative regulation of supramolecular fiber organization | GO_BiologicalProcess-_11.01.2018_00h00 | 0.03 | 0.48 | 0.00 | 0.01 | [4, 5, 6] | Group18 | 4.12 | 4.00 | [MTPN, PICK1, STMN1, STMN2] |
| GO:1902905 | positive regulation of supramolecular fiber organization | GO_BiologicalProcess-_11.01.2018_00h00 | 0.00 | 0.03 | 0.00 | 0.01 | [4, 5, 6] | Group18 | 5.59 | 8.00 | [ARF6, ARPC2, CFL1, PFN1, PICK1, PROX1, RPS3, STMN2] |
| GO:0031333 | negative regulation of protein complex assembly | GO_BiologicalProcess-_11.01.2018_00h00 | 0.01 | 0.33 | 0.00 | 0.01 | [4, 5, 6, 7] | Group18 | 5.38 | 5.00 | [HMGB1, LMO4, MTPN, STMN1, STMN2] |
| GO:0030832 | regulation of actin filament length | GO_BiologicalProcess-_11.01.2018_00h00 | 0.01 | 0.33 | 0.00 | 0.01 | [4, 5, 6, 7, 8] | Group18 | 4.55 | 6.00 | [ARF6, ARPC2, CFL1, MTPN, PFN1, PICK1] |
| GO:0032271 | regulation of protein polymerization | GO_BiologicalProcess-_11.01.2018_00h00 | 0.00 | 0.04 | 0.00 | 0.01 | [4, 5, 6, 7, 8] | Group18 | 5.33 | 8.00 | [ARF6, ARPC2, MTPN, PFN1, PICK1, RPS3, STMN1, STMN2] |
| GO:0043624 | cellular protein complex disassembly | GO_BiologicalProcess-_11.01.2018_00h00 | 0.03 | 0.53 | 0.00 | 0.01 | [6] | Group18 | 4.21 | 4.00 | [CFL1, MTPN, STMN1, STMN2] |
| GO:0046785 | microtubule polymerization | GO_BiologicalProcess-_11.01.2018_00h00 | 0.03 | 0.45 | 0.00 | 0.01 | [4, 5, 7, 8] | Group18 | 5.36 | 3.00 | [RPS3, STMN1, STMN2] |
| GO:0051495 | positive regulation of cytoskeleton organization | GO_BiologicalProcess-_11.01.2018_00h00 | 0.00 | 0.06 | 0.00 | 0.01 | [5, 6, 7] | Group18 | 5.06 | 8.00 | [ARF6, ARPC2, CFL1, PFN1, PICK1, PROX1, RPS3, STMN2] |
| GO:0031110 | regulation of microtubule polymerization or depolymerization | GO_BiologicalProcess-_11.01.2018_00h00 | 0.03 | 0.45 | 0.00 | 0.01 | [5, 6, 7, 8] | Group18 | 5.36 | 3.00 | [RPS3, STMN1, STMN2] |
| GO:0032273 | positive regulation of protein polymerization | GO_BiologicalProcess-_11.01.2018_00h00 | 0.03 | 0.58 | 0.00 | 0.01 | [4, 5, 6, 7, 8, 9] | Group18 | 4.35 | 4.00 | [ARF6, ARPC2, PICK1, RPS3] |
| GO:1901879 | regulation of protein depolymerization | GO_BiologicalProcess-_11.01.2018_00h00 | 0.04 | 0.36 | 0.00 | 0.01 | [5, 6, 7, 8] | Group18 | 5.00 | 3.00 | [CFL1, MTPN, STMN2] |
| GO:0008064 | regulation of actin polymerization or depolymerization | GO_BiologicalProcess-_11.01.2018_00h00 | 0.01 | 0.33 | 0.00 | 0.01 | [5, 6, 7, 8, 9] | Group18 | 4.55 | 6.00 | [ARF6, ARPC2, CFL1, MTPN, PFN1, PICK1] |
| GO:0031113 | regulation of microtubule polymerization | GO_BiologicalProcess-_11.01.2018_00h00 | 0.01 | 0.38 | 0.00 | 0.01 | [5, 6, 7, 8, 9] | Group18 | 9.09 | 3.00 | [RPS3, STMN1, STMN2] |
| GO:0032272 | negative regulation of protein polymerization | GO_BiologicalProcess-_11.01.2018_00h00 | 0.02 | 0.56 | 0.00 | 0.01 | [5, 6, 7, 8, 9] | Group18 | 6.00 | 3.00 | [MTPN, STMN1, STMN2] |
| GO:0051261 | protein depolymerization | GO_BiologicalProcess-_11.01.2018_00h00 | 0.02 | 0.51 | 0.00 | 0.01 | [7] | Group18 | 5.00 | 4.00 | [CFL1, MTPN, STMN1, STMN2] |
| GO:0030833 | regulation of actin filament polymerization | GO_BiologicalProcess-_11.01.2018_00h00 | 0.01 | 0.49 | 0.00 | 0.01 | [5, 6, 7, 8, 9, 10] | Group18 | 4.31 | 5.00 | [ARF6, ARPC2, MTPN, PFN1, PICK1] |
| GO:0030838 | positive regulation of actin filament polymerization | GO_BiologicalProcess-_11.01.2018_00h00 | 0.06 | 0.06 | 0.00 | 0.01 | [5, 6, 7, 8, 9, 10, 11] | Group18 | 4.11 | 3.00 | [ARF6, ARPC2, PICK1] |
| GO:0022904 | respiratory electron transport chain | GO_BiologicalProcess-_11.01.2018_00h00 | 0.01 | 0.50 | 0.01 | 0.07 | [4, 5, 6] | Group19 | 5.33 | 4.00 | [COX1, ND2, ND4, ND5] |
| GO:0006119 | oxidative phosphorylation | GO_BiologicalProcess-_11.01.2018_00h00 | 0.01 | 0.48 | 0.01 | 0.07 | [4, 5, 6, 8, 9, 10, 11] | Group19 | 5.56 | 4.00 | [COX1, ND2, ND4, ND5] |
| GO:0042773 | ATP synthesis coupled electron transport | GO_BiologicalProcess-_11.01.2018_00h00 | 0.00 | 0.32 | 0.01 | 0.07 | [5, 6, 7, 9, 10, 11, 12] | Group19 | 7.02 | 4.00 | [COX1, ND2, ND4, ND5] |
| GO:0042775 | mitochondrial ATP synthesis coupled electron transport | GO_BiologicalProcess-_11.01.2018_00h00 | 0.03 | 0.57 | 0.01 | 0.07 | [6, 7, 8, 10, 11, 12, 13] | Group19 | 5.66 | 3.00 | [COX1, ND2, ND4] |
| GO:0030010 | establishment of cell polarity | GO_BiologicalProcess-_11.01.2018_00h00 | 0.01 | 0.36 | 0.00 | 0.01 | [3] | Group20 | 5.10 | 5.00 | [ARF6, CFL1, HES1, RACK1, SPRY2] |
| GO:0051099 | positive regulation of binding | GO_BiologicalProcess-_11.01.2018_00h00 | 0.01 | 0.36 | 0.00 | 0.01 | [4] | Group20 | 4.38 | 6.00 | [HAND2, HES1, HIST1H2BA, HMGB1, RPL11, STMN1] |
| GO:0051101 | regulation of DNA binding | GO_BiologicalProcess-_11.01.2018_00h00 | 0.02 | 0.48 | 0.00 | 0.01 | [4] | Group20 | 4.88 | 4.00 | [HAND2, HES1, HMGB1, TAF10] |
| GO:0048864 | stem cell development | GO_BiologicalProcess-_11.01.2018_00h00 | 0.03 | 0.39 | 0.00 | 0.01 | [4, 5] | Group20 | 5.17 | 3.00 | [CFL1, HAND2, HES1] |
| GO:0061008 | hepaticobiliary system development | GO_BiologicalProcess-_11.01.2018_00h00 | 0.01 | 0.38 | 0.00 | 0.01 | [4, 5] | Group20 | 6.25 | 4.00 | [ARF6, HES1, PROX1, TAF10] |
| GO:0043388 | positive regulation of DNA binding | GO_BiologicalProcess-_11.01.2018_00h00 | 0.01 | 0.48 | 0.00 | 0.01 | [5] | Group20 | 7.69 | 3.00 | [HAND2, HES1, HMGB1] |
| GO:0048483 | autonomic nervous system development | GO_BiologicalProcess-_11.01.2018_00h00 | 0.01 | 0.48 | 0.00 | 0.01 | [4, 5, 6] | Group20 | 7.69 | 3.00 | [EGR2, HAND2, HES1] |
| GO:0048645 | animal organ formation | GO_BiologicalProcess-_11.01.2018_00h00 | 0.03 | 0.41 | 0.00 | 0.01 | [3, 4, 5, 6, 7] | Group20 | 5.26 | 3.00 | [HAND2, HES1, HOXC11] |
| GO:0003279 | cardiac septum development | GO_BiologicalProcess-_11.01.2018_00h00 | 0.02 | 0.50 | 0.00 | 0.01 | [3, 4, 6, 7, 8] | Group20 | 5.06 | 4.00 | [HES1, LMO4, LUZP1, PROX1] |
| GO:0048538 | thymus development | GO_BiologicalProcess-_11.01.2018_00h00 | 0.00 | 0.12 | 0.00 | 0.01 | [4, 5, 6, 7] | Group20 | 9.52 | 4.00 | [BCL2L11, HAND2, HES1, LMO4] |
| GO:0001889 | liver development | GO_BiologicalProcess-_11.01.2018_00h00 | 0.01 | 0.36 | 0.00 | 0.01 | [5, 6, 7] | Group20 | 6.56 | 4.00 | [ARF6, HES1, PROX1, TAF10] |
| GO:0003156 | regulation of animal organ formation | GO_BiologicalProcess-_11.01.2018_00h00 | 0.01 | 0.36 | 0.00 | 0.01 | [4, 5, 6, 7, 8] | Group20 | 9.38 | 3.00 | [HAND2, HES1, HOXC11] |
| GO:0014031 | mesenchymal cell development | GO_BiologicalProcess-_11.01.2018_00h00 | 0.03 | 0.38 | 0.00 | 0.01 | [4, 5, 6, 7, 8] | Group20 | 5.08 | 3.00 | [CFL1, HAND2, HES1] |
| GO:0042733 | embryonic digit morphogenesis | GO_BiologicalProcess-_11.01.2018_00h00 | 0.03 | 0.41 | 0.00 | 0.01 | [4, 5, 6, 7, 8] | Group20 | 5.26 | 3.00 | [BCL2L11, HAND2, HOXC11] |
| GO:1903707 | negative regulation of hemopoiesis | GO_BiologicalProcess-_11.01.2018_00h00 | 0.03 | 0.49 | 0.00 | 0.01 | [3, 4, 5, 6, 7, 8, 9] | Group20 | 4.17 | 4.00 | [CD74, HES1, HMGB1, NRARP] |
| GO:0021575 | hindbrain morphogenesis | GO_BiologicalProcess-_11.01.2018_00h00 | 0.01 | 0.48 | 0.00 | 0.01 | [3, 4, 6, 7, 8, 9] | Group20 | 7.69 | 3.00 | [CBLN1, HES1, PROX1] |
| GO:0003231 | cardiac ventricle development | GO_BiologicalProcess-_11.01.2018_00h00 | 0.01 | 0.36 | 0.00 | 0.01 | [4, 6, 7, 8] | Group20 | 5.15 | 5.00 | [HAND2, HES1, LMO4, LUZP1, PROX1] |
| GO:0003281 | ventricular septum development | GO_BiologicalProcess-_11.01.2018_00h00 | 0.00 | 0.24 | 0.00 | 0.01 | [4, 5, 7, 8, 9] | Group20 | 7.84 | 4.00 | [HES1, LMO4, LUZP1, PROX1] |
| GO:0014033 | neural crest cell differentiation | GO_BiologicalProcess-_11.01.2018_00h00 | 0.04 | 0.35 | 0.00 | 0.01 | [5, 6, 7, 8] | Group20 | 4.84 | 3.00 | [CFL1, HAND2, HES1] |
| GO:0060840 | artery development | GO_BiologicalProcess-_11.01.2018_00h00 | 0.01 | 0.39 | 0.00 | 0.01 | [4, 6, 7, 8, 9] | Group20 | 6.15 | 4.00 | [HAND2, HES1, LUZP1, PROX1] |
| GO:0014032 | neural crest cell development | GO_BiologicalProcess-_11.01.2018_00h00 | 0.03 | 0.51 | 0.00 | 0.01 | [5, 6, 7, 8, 9] | Group20 | 5.45 | 3.00 | [CFL1, HAND2, HES1] |
| GO:0048844 | artery morphogenesis | GO_BiologicalProcess-_11.01.2018_00h00 | 0.02 | 0.56 | 0.00 | 0.01 | [4, 5, 7, 8, 9, 10] | Group20 | 6.00 | 3.00 | [HAND2, HES1, PROX1] |
| GO:0042102 | positive regulation of T cell proliferation | GO_BiologicalProcess-_11.01.2018_00h00 | 0.06 | 0.06 | 0.00 | 0.01 | [6, 7, 8, 9] | Group20 | 4.11 | 3.00 | [HES1, HMGB1, RPS3] |
| GO:0022613 | ribonucleoprotein complex biogenesis | GO_BiologicalProcess-_11.01.2018_00h00 | 0.00 | 0.00 | 0.00 | 0.00 | [3] | Group21 | 7.30 | 26.00 | [RPL10, RPL11, RPL13A, RPL14, RPL26, RPL3, RPL34, RPL35, RPL35A, RPL38, RPL5, RPLP0, RPS10, RPS15, RPS17, RPS19, RPS23, RPS24, RPS27, RPS28, RPS5, RPS7, RPS8, RRP1B, SF3B1, SNRPG] |
| GO:0042254 | ribosome biogenesis | GO_BiologicalProcess-_11.01.2018_00h00 | 0.00 | 0.00 | 0.00 | 0.00 | [4] | Group21 | 9.57 | 22.00 | [RPL10, RPL11, RPL14, RPL26, RPL3, RPL34, RPL35, RPL35A, RPL38, RPL5, RPLP0, RPS10, RPS15, RPS17, RPS19, RPS24, RPS27, RPS28, RPS5, RPS7, RPS8, RRP1B] |
| GO:0043603 | cellular amide metabolic process | GO_BiologicalProcess-_11.01.2018_00h00 | 0.00 | 0.00 | 0.00 | 0.00 | [4] | Group21 | 7.16 | 53.00 | [DDX5, EEF1A1, EEF1G, EIF1, EIF4A1, ELOB, FAU, HNRNPA2B1, RACK1, RPL10, RPL11, RPL13, RPL13A, RPL14, RPL18, RPL19, RPL23, RPL26, RPL27A, RPL3, RPL30, RPL31, RPL32, RPL34, RPL35, RPL35A, RPL36AL, RPL37, RPL37A, RPL38, RPL5, RPL8, RPLP1, RPLP2, RPS11, RPS12, RPS14, RPS15, RPS17, RPS19, RPS2, RPS20, RPS23, RPS24, RPS27, RPS27A, RPS28, RPS3, RPS5, RPS7, RPS8, RPS9, UBA52] |
| GO:0071826 | ribonucleoprotein complex subunit organization | GO_BiologicalProcess-_11.01.2018_00h00 | 0.00 | 0.00 | 0.00 | 0.00 | [4] | Group21 | 8.25 | 16.00 | [RPL10, RPL11, RPL13A, RPL3, RPL38, RPL5, RPS10, RPS15, RPS17, RPS19, RPS23, RPS27, RPS28, RPS5, SF3B1, SNRPG] |
| GO:1901566 | organonitrogen compound biosynthetic process | GO_BiologicalProcess-_11.01.2018_00h00 | 0.00 | 0.00 | 0.00 | 0.00 | [4] | Group21 | 4.67 | 63.00 | [AKAP12, ATP6, DDX5, EEF1A1, EEF1G, EIF1, EIF4A1, ELOB, FABP3, FAU, HAND2, HNRNPA2B1, MC4R, MGAT1, MUSTN1, OAZ1, P2RY13, RACK1, RPL10, RPL11, RPL13, RPL13A, RPL14, RPL18, RPL19, RPL23, RPL26, RPL27A, RPL3, RPL30, RPL31, RPL32, RPL34, RPL35, RPL35A, RPL36AL, RPL37, RPL37A, RPL38, RPL5, RPL8, RPLP1, RPLP2, RPS11, RPS12, RPS14, RPS15, RPS17, RPS19, RPS2, RPS20, RPS23, RPS24, RPS27, RPS27A, RPS28, RPS3, RPS5, RPS7, RPS8, RPS9, TRIB2, UBA52] |
| GO:0006518 | peptide metabolic process | GO_BiologicalProcess-_11.01.2018_00h00 | 0.00 | 0.00 | 0.00 | 0.00 | [4, 5] | Group21 | 8.43 | 53.00 | [DDX5, EEF1A1, EEF1G, EIF1, EIF4A1, ELOB, FAU, HNRNPA2B1, RACK1, RPL10, RPL11, RPL13, RPL13A, RPL14, RPL18, RPL19, RPL23, RPL26, RPL27A, RPL3, RPL30, RPL31, RPL32, RPL34, RPL35, RPL35A, RPL36AL, RPL37, RPL37A, RPL38, RPL5, RPL8, RPLP1, RPLP2, RPS11, RPS12, RPS14, RPS15, RPS17, RPS19, RPS2, RPS20, RPS23, RPS24, RPS27, RPS27A, RPS28, RPS3, RPS5, RPS7, RPS8, RPS9, UBA52] |
| GO:0042273 | ribosomal large subunit biogenesis | GO_BiologicalProcess-_11.01.2018_00h00 | 0.00 | 0.00 | 0.00 | 0.00 | [4, 5] | Group21 | 13.43 | 9.00 | [RPL10, RPL11, RPL14, RPL26, RPL3, RPL35, RPL35A, RPL38, RPL5] |
| GO:0042274 | ribosomal small subunit biogenesis | GO_BiologicalProcess-_11.01.2018_00h00 | 0.00 | 0.00 | 0.00 | 0.00 | [4, 5] | Group21 | 16.92 | 11.00 | [RPL38, RPS10, RPS15, RPS17, RPS19, RPS24, RPS27, RPS28, RPS5, RPS7, RPS8] |
| GO:0022618 | ribonucleoprotein complex assembly | GO_BiologicalProcess-_11.01.2018_00h00 | 0.00 | 0.00 | 0.00 | 0.00 | [4, 5, 6] | Group21 | 8.65 | 16.00 | [RPL10, RPL11, RPL13A, RPL3, RPL38, RPL5, RPS10, RPS15, RPS17, RPS19, RPS23, RPS27, RPS28, RPS5, SF3B1, SNRPG] |
| GO:0043604 | amide biosynthetic process | GO_BiologicalProcess-_11.01.2018_00h00 | 0.00 | 0.00 | 0.00 | 0.00 | [5] | Group21 | 9.01 | 53.00 | [DDX5, EEF1A1, EEF1G, EIF1, EIF4A1, ELOB, FAU, HNRNPA2B1, RACK1, RPL10, RPL11, RPL13, RPL13A, RPL14, RPL18, RPL19, RPL23, RPL26, RPL27A, RPL3, RPL30, RPL31, RPL32, RPL34, RPL35, RPL35A, RPL36AL, RPL37, RPL37A, RPL38, RPL5, RPL8, RPLP1, RPLP2, RPS11, RPS12, RPS14, RPS15, RPS17, RPS19, RPS2, RPS20, RPS23, RPS24, RPS27, RPS27A, RPS28, RPS3, RPS5, RPS7, RPS8, RPS9, UBA52] |
| GO:0043043 | peptide biosynthetic process | GO_BiologicalProcess-_11.01.2018_00h00 | 0.00 | 0.00 | 0.00 | 0.00 | [5, 6] | Group21 | 10.04 | 53.00 | [DDX5, EEF1A1, EEF1G, EIF1, EIF4A1, ELOB, FAU, HNRNPA2B1, RACK1, RPL10, RPL11, RPL13, RPL13A, RPL14, RPL18, RPL19, RPL23, RPL26, RPL27A, RPL3, RPL30, RPL31, RPL32, RPL34, RPL35, RPL35A, RPL36AL, RPL37, RPL37A, RPL38, RPL5, RPL8, RPLP1, RPLP2, RPS11, RPS12, RPS14, RPS15, RPS17, RPS19, RPS2, RPS20, RPS23, RPS24, RPS27, RPS27A, RPS28, RPS3, RPS5, RPS7, RPS8, RPS9, UBA52] |
| GO:0006412 | translation | GO_BiologicalProcess-_11.01.2018_00h00 | 0.00 | 0.00 | 0.00 | 0.00 | [5, 6, 7] | Group21 | 10.35 | 53.00 | [DDX5, EEF1A1, EEF1G, EIF1, EIF4A1, ELOB, FAU, HNRNPA2B1, RACK1, RPL10, RPL11, RPL13, RPL13A, RPL14, RPL18, RPL19, RPL23, RPL26, RPL27A, RPL3, RPL30, RPL31, RPL32, RPL34, RPL35, RPL35A, RPL36AL, RPL37, RPL37A, RPL38, RPL5, RPL8, RPLP1, RPLP2, RPS11, RPS12, RPS14, RPS15, RPS17, RPS19, RPS2, RPS20, RPS23, RPS24, RPS27, RPS27A, RPS28, RPS3, RPS5, RPS7, RPS8, RPS9, UBA52] |
| GO:0042255 | ribosome assembly | GO_BiologicalProcess-_11.01.2018_00h00 | 0.00 | 0.00 | 0.00 | 0.00 | [5, 6, 7] | Group21 | 22.22 | 12.00 | [RPL10, RPL11, RPL3, RPL38, RPL5, RPS10, RPS15, RPS17, RPS19, RPS27, RPS28, RPS5] |
| GO:0000027 | ribosomal large subunit assembly | GO_BiologicalProcess-_11.01.2018_00h00 | 0.00 | 0.00 | 0.00 | 0.00 | [5, 6, 7, 8] | Group21 | 17.24 | 5.00 | [RPL10, RPL11, RPL3, RPL38, RPL5] |
| GO:0000028 | ribosomal small subunit assembly | GO_BiologicalProcess-_11.01.2018_00h00 | 0.00 | 0.00 | 0.00 | 0.00 | [5, 6, 7, 8] | Group21 | 50.00 | 8.00 | [RPL38, RPS10, RPS15, RPS17, RPS19, RPS27, RPS28, RPS5] |
| GO:0002181 | cytoplasmic translation | GO_BiologicalProcess-_11.01.2018_00h00 | 0.00 | 0.00 | 0.00 | 0.00 | [6, 7, 8] | Group21 | 20.00 | 12.00 | [RPL13A, RPL26, RPL27A, RPL30, RPL31, RPL35A, RPL8, RPLP1, RPLP2, RPS20, RPS3, RPS7] |
| GO:0016072 | rRNA metabolic process | GO_BiologicalProcess-_11.01.2018_00h00 | 0.00 | 0.00 | 0.00 | 0.00 | [6, 7, 8] | Group21 | 6.29 | 11.00 | [RPL11, RPL14, RPL26, RPL35, RPS17, RPS24, RPS27, RPS28, RPS7, RPS8, RRP1B] |
| GO:0034470 | ncRNA processing | GO_BiologicalProcess-_11.01.2018_00h00 | 0.00 | 0.01 | 0.00 | 0.00 | [6, 7, 8] | Group21 | 4.36 | 12.00 | [HNRNPA2B1, RPL11, RPL14, RPL26, RPL35, RPS17, RPS24, RPS27, RPS28, RPS7, RPS8, RRP1B] |
| GO:0006364 | rRNA processing | GO_BiologicalProcess-_11.01.2018_00h00 | 0.00 | 0.00 | 0.00 | 0.00 | [5, 7, 8, 9] | Group21 | 7.28 | 11.00 | [RPL11, RPL14, RPL26, RPL35, RPS17, RPS24, RPS27, RPS28, RPS7, RPS8, RRP1B] |
| GO:0030490 | maturation of SSU-rRNA | GO_BiologicalProcess-_11.01.2018_00h00 | 0.02 | 0.51 | 0.00 | 0.00 | [5, 6, 8, 9, 10] | Group21 | 6.38 | 3.00 | [RPS24, RPS28, RPS8] |
| GO:0001935 | endothelial cell proliferation | GO_BiologicalProcess-_11.01.2018_00h00 | 0.03 | 0.55 | 0.00 | 0.00 | [3] | Group22 | 4.30 | 4.00 | [HMGB1, NRARP, PROX1, THAP1] |
| GO:0001776 | leukocyte homeostasis | GO_BiologicalProcess-_11.01.2018_00h00 | 0.06 | 0.11 | 0.00 | 0.00 | [2, 5] | Group22 | 4.17 | 3.00 | [BCL2L11, CD74, HMGB1] |
| GO:0051099 | positive regulation of binding | GO_BiologicalProcess-_11.01.2018_00h00 | 0.01 | 0.36 | 0.00 | 0.00 | [4] | Group22 | 4.38 | 6.00 | [HAND2, HES1, HIST1H2BA, HMGB1, RPL11, STMN1] |
| GO:0051101 | regulation of DNA binding | GO_BiologicalProcess-_11.01.2018_00h00 | 0.02 | 0.48 | 0.00 | 0.00 | [4] | Group22 | 4.88 | 4.00 | [HAND2, HES1, HMGB1, TAF10] |
| GO:0051881 | regulation of mitochondrial membrane potential | GO_BiologicalProcess-_11.01.2018_00h00 | 0.00 | 0.24 | 0.00 | 0.00 | [4] | Group22 | 7.84 | 4.00 | [MLLT11, RACK1, UBA52, UBB] |
| GO:0001836 | release of cytochrome c from mitochondria | GO_BiologicalProcess-_11.01.2018_00h00 | 0.01 | 0.50 | 0.00 | 0.00 | [4, 5, 6] | Group22 | 7.32 | 3.00 | [APOPT1, BCL2L11, MLLT11] |
| GO:0008637 | apoptotic mitochondrial changes | GO_BiologicalProcess-_11.01.2018_00h00 | 0.06 | 0.06 | 0.00 | 0.00 | [5] | Group22 | 4.11 | 3.00 | [APOPT1, BCL2L11, MLLT11] |
| GO:0042770 | signal transduction in response to DNA damage | GO_BiologicalProcess-_11.01.2018_00h00 | 0.05 | 0.24 | 0.00 | 0.00 | [4, 5, 6] | Group22 | 4.41 | 3.00 | [CD74, DDX5, RPL26] |
| GO:0043388 | positive regulation of DNA binding | GO_BiologicalProcess-_11.01.2018_00h00 | 0.01 | 0.48 | 0.00 | 0.00 | [5] | Group22 | 7.69 | 3.00 | [HAND2, HES1, HMGB1] |
| GO:0072331 | signal transduction by p53 class mediator | GO_BiologicalProcess-_11.01.2018_00h00 | 0.00 | 0.04 | 0.00 | 0.00 | [4, 5, 6] | Group22 | 6.14 | 7.00 | [CD74, DDX5, RPL11, RPL26, RPS7, UBA52, UBB] |
| GO:0097193 | intrinsic apoptotic signaling pathway | GO_BiologicalProcess-_11.01.2018_00h00 | 0.00 | 0.00 | 0.00 | 0.00 | [4, 5, 6] | Group22 | 5.06 | 12.00 | [APOPT1, BCL2L11, CD74, DDX5, MLLT11, RACK1, RPL11, RPL26, RPS3, RPS7, UBA52, UBB] |
| GO:2001022 | positive regulation of response to DNA damage stimulus | GO_BiologicalProcess-_11.01.2018_00h00 | 0.01 | 0.41 | 0.00 | 0.00 | [3, 4, 5, 6, 7] | Group22 | 5.97 | 4.00 | [DDX5, HMGB1, RPL26, RPS3] |
| GO:0008630 | intrinsic apoptotic signaling pathway in response to DNA damage | GO_BiologicalProcess-_11.01.2018_00h00 | 0.02 | 0.52 | 0.00 | 0.00 | [5, 6, 7] | Group22 | 4.65 | 4.00 | [BCL2L11, CD74, RPL26, RPS3] |
| GO:0030330 | DNA damage response, signal transduction by p53 class mediator | GO_BiologicalProcess-_11.01.2018_00h00 | 0.03 | 0.51 | 0.00 | 0.00 | [5, 6, 7] | Group22 | 5.45 | 3.00 | [CD74, DDX5, RPL26] |
| GO:0072332 | intrinsic apoptotic signaling pathway by p53 class mediator | GO_BiologicalProcess-_11.01.2018_00h00 | 0.00 | 0.00 | 0.00 | 0.00 | [5, 6, 7] | Group22 | 10.29 | 7.00 | [CD74, DDX5, RPL11, RPL26, RPS7, UBA52, UBB] |
| GO:1901796 | regulation of signal transduction by p53 class mediator | GO_BiologicalProcess-_11.01.2018_00h00 | 0.00 | 0.00 | 0.00 | 0.00 | [5, 6, 7] | Group22 | 13.46 | 7.00 | [CD74, DDX5, RPL11, RPL26, RPS7, UBA52, UBB] |
| GO:1903707 | negative regulation of hemopoiesis | GO_BiologicalProcess-_11.01.2018_00h00 | 0.03 | 0.49 | 0.00 | 0.00 | [3, 4, 5, 6, 7, 8, 9] | Group22 | 4.17 | 4.00 | [CD74, HES1, HMGB1, NRARP] |
| GO:2001235 | positive regulation of apoptotic signaling pathway | GO_BiologicalProcess-_11.01.2018_00h00 | 0.00 | 0.00 | 0.00 | 0.00 | [4, 5, 6, 7, 8] | Group22 | 7.81 | 10.00 | [APOPT1, BCL2L11, MLLT11, RACK1, RPL11, RPL26, RPS3, RPS7, UBA52, UBB] |
| GO:0043516 | regulation of DNA damage response, signal transduction by p53 class mediator | GO_BiologicalProcess-_11.01.2018_00h00 | 0.00 | 0.27 | 0.00 | 0.00 | [5, 6, 7, 8] | Group22 | 11.11 | 3.00 | [CD74, DDX5, RPL26] |
| GO:0090199 | regulation of release of cytochrome c from mitochondria | GO_BiologicalProcess-_11.01.2018_00h00 | 0.01 | 0.36 | 0.00 | 0.00 | [5, 6, 7, 8] | Group22 | 9.68 | 3.00 | [APOPT1, BCL2L11, MLLT11] |
| GO:1901798 | positive regulation of signal transduction by p53 class mediator | GO_BiologicalProcess-_11.01.2018_00h00 | 0.00 | 0.00 | 0.00 | 0.00 | [5, 6, 7, 8] | Group22 | 33.33 | 6.00 | [DDX5, RPL11, RPL26, RPS7, UBA52, UBB] |
| GO:2001242 | regulation of intrinsic apoptotic signaling pathway | GO_BiologicalProcess-_11.01.2018_00h00 | 0.00 | 0.02 | 0.00 | 0.00 | [5, 6, 7, 8] | Group22 | 6.06 | 8.00 | [CD74, RACK1, RPL11, RPL26, RPS3, RPS7, UBA52, UBB] |
| GO:0090200 | positive regulation of release of cytochrome c from mitochondria | GO_BiologicalProcess-_11.01.2018_00h00 | 0.00 | 0.10 | 0.00 | 0.00 | [5, 6, 7, 8, 9] | Group22 | 16.67 | 3.00 | [APOPT1, BCL2L11, MLLT11] |
| GO:1902106 | negative regulation of leukocyte differentiation | GO_BiologicalProcess-_11.01.2018_00h00 | 0.05 | 0.28 | 0.00 | 0.00 | [4, 5, 6, 7, 8, 9, 10] | Group22 | 4.48 | 3.00 | [CD74, HMGB1, NRARP] |
| GO:1902229 | regulation of intrinsic apoptotic signaling pathway in response to DNA damage | GO_BiologicalProcess-_11.01.2018_00h00 | 0.01 | 0.34 | 0.00 | 0.00 | [5, 6, 7, 8, 9] | Group22 | 10.00 | 3.00 | [CD74, RPL26, RPS3] |
| GO:1903050 | regulation of proteolysis involved in cellular protein catabolic process | GO_BiologicalProcess-_11.01.2018_00h00 | 0.00 | 0.23 | 0.00 | 0.00 | [6, 7, 8] | Group22 | 4.38 | 7.00 | [MGC137055, RACK1, RPL11, RPS7, TRIB2, UBA52, UBB] |
| GO:2001244 | positive regulation of intrinsic apoptotic signaling pathway | GO_BiologicalProcess-_11.01.2018_00h00 | 0.00 | 0.00 | 0.00 | 0.00 | [5, 6, 7, 8, 9] | Group22 | 14.29 | 7.00 | [RACK1, RPL11, RPL26, RPS3, RPS7, UBA52, UBB] |
| GO:0010952 | positive regulation of peptidase activity | GO_BiologicalProcess-_11.01.2018_00h00 | 0.01 | 0.49 | 0.00 | 0.00 | [6, 7, 8, 9] | Group22 | 4.50 | 5.00 | [APOPT1, BCL2L11, HMGB1, RACK1, RPS3] |
| GO:0042102 | positive regulation of T cell proliferation | GO_BiologicalProcess-_11.01.2018_00h00 | 0.06 | 0.06 | 0.00 | 0.00 | [6, 7, 8, 9] | Group22 | 4.11 | 3.00 | [HES1, HMGB1, RPS3] |
| GO:0061136 | regulation of proteasomal protein catabolic process | GO_BiologicalProcess-_11.01.2018_00h00 | 0.01 | 0.40 | 0.00 | 0.00 | [6, 7, 8, 9] | Group22 | 4.20 | 6.00 | [MGC137055, RACK1, RPL11, TRIB2, UBA52, UBB] |
| GO:1902253 | regulation of intrinsic apoptotic signaling pathway by p53 class mediator | GO_BiologicalProcess-_11.01.2018_00h00 | 0.00 | 0.00 | 0.00 | 0.00 | [6, 7, 8, 9] | Group22 | 26.09 | 6.00 | [CD74, RPL11, RPL26, RPS7, UBA52, UBB] |
| GO:1903321 | negative regulation of protein modification by small protein conjugation or removal | GO_BiologicalProcess-_11.01.2018_00h00 | 0.04 | 0.35 | 0.00 | 0.00 | [6, 7, 8, 9] | Group22 | 4.62 | 3.00 | [RPL11, RPS3, RPS7] |
| GO:0045620 | negative regulation of lymphocyte differentiation | GO_BiologicalProcess-_11.01.2018_00h00 | 0.01 | 0.38 | 0.00 | 0.00 | [5, 6, 7, 8, 9, 10, 11] | Group22 | 8.82 | 3.00 | [CD74, HMGB1, NRARP] |
| GO:1902255 | positive regulation of intrinsic apoptotic signaling pathway by p53 class mediator | GO_BiologicalProcess-_11.01.2018_00h00 | 0.00 | 0.00 | 0.00 | 0.00 | [6, 7, 8, 9, 10] | Group22 | 83.33 | 5.00 | [RPL11, RPL26, RPS7, UBA52, UBB] |
| GO:0010950 | positive regulation of endopeptidase activity | GO_BiologicalProcess-_11.01.2018_00h00 | 0.01 | 0.36 | 0.00 | 0.00 | [7, 8, 9, 10] | Group22 | 5.15 | 5.00 | [APOPT1, BCL2L11, HMGB1, RACK1, RPS3] |
| GO:0045580 | regulation of T cell differentiation | GO_BiologicalProcess-_11.01.2018_00h00 | 0.03 | 0.53 | 0.00 | 0.00 | [6, 7, 8, 9, 10, 11] | Group22 | 4.21 | 4.00 | [CD74, DUSP10, HMGB1, NRARP] |
| GO:0031397 | negative regulation of protein ubiquitination | GO_BiologicalProcess-_11.01.2018_00h00 | 0.03 | 0.41 | 0.00 | 0.00 | [7, 8, 9, 10, 11] | Group22 | 5.26 | 3.00 | [RPL11, RPS3, RPS7] |
| GO:0032434 | regulation of proteasomal ubiquitin-dependent protein catabolic process | GO_BiologicalProcess-_11.01.2018_00h00 | 0.03 | 0.49 | 0.00 | 0.00 | [7, 8, 9, 10, 11] | Group22 | 4.17 | 4.00 | [MGC137055, RACK1, RPL11, TRIB2] |
| GO:0043280 | positive regulation of cysteine-type endopeptidase activity involved in apoptotic process | GO_BiologicalProcess-_11.01.2018_00h00 | 0.00 | 0.18 | 0.00 | 0.00 | [6, 7, 8, 9, 10, 11, 12] | Group22 | 6.49 | 5.00 | [APOPT1, BCL2L11, HMGB1, RACK1, RPS3] |
| GO:0045581 | negative regulation of T cell differentiation | GO_BiologicalProcess-_11.01.2018_00h00 | 0.00 | 0.32 | 0.00 | 0.00 | [6, 7, 8, 9, 10, 11, 12] | Group22 | 10.34 | 3.00 | [CD74, HMGB1, NRARP] |
| GO:2001056 | positive regulation of cysteine-type endopeptidase activity | GO_BiologicalProcess-_11.01.2018_00h00 | 0.00 | 0.25 | 0.00 | 0.00 | [8, 9, 10, 11] | Group22 | 5.88 | 5.00 | [APOPT1, BCL2L11, HMGB1, RACK1, RPS3] |
| GO:0016363 | nuclear matrix | GO_CellularComponent-_11.01.2018_00h00 | 0.04 | 0.35 | 0.04 | 0.13 | [4, 5, 6, 7, 8, 9, 10, 11] | Group01 | 4.62 | 3.00 | [CFL1, PSMA6, RAD21] |
| GO:0005912 | adherens junction | GO_CellularComponent-_11.01.2018_00h00 | 0.00 | 0.00 | 0.00 | 0.00 | [3] | Group09 | 5.50 | 22.00 | [ACTB, AKAP12, ARF6, ARPC2, CFL1, MARCKS, RPL18, RPL30, RPL37A, RPLP0, RPLP1, RPLP2, RPS10, RPS11, RPS17, RPS3, RPS5, RPS7, RPS8, RPS9, YWHAB, YWHAZ] |
| GO:0005924 | cell-substrate adherens junction | GO_CellularComponent-_11.01.2018_00h00 | 0.00 | 0.00 | 0.00 | 0.00 | [3, 4] | Group09 | 6.94 | 22.00 | [ACTB, AKAP12, ARF6, ARPC2, CFL1, MARCKS, RPL18, RPL30, RPL37A, RPLP0, RPLP1, RPLP2, RPS10, RPS11, RPS17, RPS3, RPS5, RPS7, RPS8, RPS9, YWHAB, YWHAZ] |
| GO:0005925 | focal adhesion | GO_CellularComponent-_11.01.2018_00h00 | 0.00 | 0.00 | 0.00 | 0.00 | [4, 5] | Group09 | 7.01 | 22.00 | [ACTB, AKAP12, ARF6, ARPC2, CFL1, MARCKS, RPL18, RPL30, RPL37A, RPLP0, RPLP1, RPLP2, RPS10, RPS11, RPS17, RPS3, RPS5, RPS7, RPS8, RPS9, YWHAB, YWHAZ] |
| GO:0030532 | small nuclear ribonucleoprotein complex | GO_CellularComponent-_11.01.2018_00h00 | 0.01 | 0.40 | 0.00 | 0.03 | [3, 4, 5, 6, 7, 8, 9] | Group13 | 6.06 | 4.00 | [LSM2, SF3B1, SNRNP40, SNRPG] |
| GO:0036464 | cytoplasmic ribonucleoprotein granule | GO_CellularComponent-_11.01.2018_00h00 | 0.01 | 0.41 | 0.00 | 0.03 | [4, 5, 6, 7, 8] | Group13 | 4.08 | 6.00 | [ACTB, LSM2, PCBP1, PSMA6, RPLP0, SNRPG] |
| GO:0071013 | catalytic step 2 spliceosome | GO_CellularComponent-_11.01.2018_00h00 | 0.00 | 0.07 | 0.00 | 0.03 | [3, 5, 6, 7, 8, 9, 10] | Group13 | 6.67 | 6.00 | [DDX5, HNRNPA2B1, LSM2, SF3B1, SNRNP40, SNRPG] |
| GO:0097525 | spliceosomal snRNP complex | GO_CellularComponent-_11.01.2018_00h00 | 0.01 | 0.36 | 0.00 | 0.03 | [4, 5, 6, 7, 8, 9, 10] | Group13 | 6.56 | 4.00 | [LSM2, SF3B1, SNRNP40, SNRPG] |
| GO:0071011 | precatalytic spliceosome | GO_CellularComponent-_11.01.2018_00h00 | 0.00 | 0.32 | 0.00 | 0.03 | [5, 6, 7, 8, 9, 10] | Group13 | 10.34 | 3.00 | [LSM2, SNRNP40, SNRPG] |
| GO:0032587 | ruffle membrane | GO_CellularComponent-_11.01.2018_00h00 | 0.03 | 0.55 | 0.01 | 0.07 | [4, 5, 6, 7, 8] | Group15 | 5.56 | 3.00 | [CFL1, RPS3, SPRY2] |
| GO:0000786 | nucleosome | GO_CellularComponent-_11.01.2018_00h00 | 0.00 | 0.05 | 0.00 | 0.03 | [3, 4, 5, 6, 7, 8, 9, 10] | Group17 | 7.14 | 6.00 | [HILS1, HIST1H2BA, PRM1, PRM2, TNP1, TNP2] |
| GO:0043073 | germ cell nucleus | GO_CellularComponent-_11.01.2018_00h00 | 0.00 | 0.10 | 0.00 | 0.03 | [5, 6, 7, 8] | Group17 | 16.67 | 3.00 | [MARCKS, TNP1, TNP2] |
| GO:0000788 | nuclear nucleosome | GO_CellularComponent-_11.01.2018_00h00 | 0.00 | 0.03 | 0.00 | 0.03 | [4, 5, 6, 7, 8, 9, 10, 11, 12, 13] | Group17 | 27.27 | 3.00 | [HIST1H2BA, TNP1, TNP2] |
| GO:0030964 | NADH dehydrogenase complex | GO_CellularComponent-_11.01.2018_00h00 | 0.00 | 0.02 | 0.01 | 0.07 | [2, 3] | Group19 | 10.64 | 5.00 | [ND1, ND2, ND3, ND4, ND5] |
| GO:0070469 | respiratory chain | GO_CellularComponent-_11.01.2018_00h00 | 0.00 | 0.05 | 0.01 | 0.07 | [2, 3] | Group19 | 7.14 | 6.00 | [COX1, ND1, ND2, ND3, ND4, ND5] |
| GO:1990204 | oxidoreductase complex | GO_CellularComponent-_11.01.2018_00h00 | 0.00 | 0.09 | 0.01 | 0.07 | [2, 3] | Group19 | 6.25 | 6.00 | [COX1, ND1, ND2, ND3, ND4, ND5] |
| GO:0098803 | respiratory chain complex | GO_CellularComponent-_11.01.2018_00h00 | 0.00 | 0.03 | 0.01 | 0.07 | [3, 4] | Group19 | 7.69 | 6.00 | [COX1, ND1, ND2, ND3, ND4, ND5] |
| GO:0045271 | respiratory chain complex I | GO_CellularComponent-_11.01.2018_00h00 | 0.00 | 0.02 | 0.01 | 0.07 | [3, 4, 5] | Group19 | 10.64 | 5.00 | [ND1, ND2, ND3, ND4, ND5] |
| GO:0098798 | mitochondrial protein complex | GO_CellularComponent-_11.01.2018_00h00 | 0.00 | 0.02 | 0.01 | 0.07 | [3, 4, 5, 6, 7, 8, 9] | Group19 | 5.88 | 8.00 | [ATP6, COX1, ND1, ND2, ND3, ND4, ND5, TOMM22] |
| GO:0044455 | mitochondrial membrane part | GO_CellularComponent-_11.01.2018_00h00 | 0.00 | 0.12 | 0.01 | 0.07 | [2, 3, 4, 5, 6, 7, 8, 9, 10, 11] | Group19 | 4.44 | 8.00 | [ATP6, COX1, ND1, ND2, ND3, ND4, ND5, TOMM22] |
| GO:0005746 | mitochondrial respiratory chain | GO_CellularComponent-_11.01.2018_00h00 | 0.00 | 0.03 | 0.01 | 0.07 | [3, 4, 5, 6, 7, 8, 9, 10, 11, 12] | Group19 | 7.89 | 6.00 | [COX1, ND1, ND2, ND3, ND4, ND5] |
| GO:0098800 | inner mitochondrial membrane protein complex | GO_CellularComponent-_11.01.2018_00h00 | 0.00 | 0.05 | 0.01 | 0.07 | [3, 4, 5, 6, 7, 8, 9, 10, 11, 12] | Group19 | 5.88 | 7.00 | [ATP6, COX1, ND1, ND2, ND3, ND4, ND5] |
| GO:0005747 | mitochondrial respiratory chain complex I | GO_CellularComponent-_11.01.2018_00h00 | 0.00 | 0.02 | 0.01 | 0.07 | [3, 4, 5, 6, 7, 8, 9, 10, 11, 12, 13] | Group19 | 10.64 | 5.00 | [ND1, ND2, ND3, ND4, ND5] |
| GO:0030529 | intracellular ribonucleoprotein complex | GO_CellularComponent-_11.01.2018_00h00 | 0.00 | 0.00 | 0.00 | 0.00 | [3, 4, 5] | Group21 | 8.58 | 61.00 | [ACTB, DDX5, FAU, HNRNPA0, HNRNPA2B1, LSM2, PABPN1, PCBP1, PSMA6, RACK1, RPL10, RPL11, RPL13, RPL13A, RPL14, RPL18, RPL19, RPL23, RPL26, RPL27A, RPL3, RPL30, RPL31, RPL32, RPL34, RPL35, RPL35A, RPL36AL, RPL37, RPL37A, RPL38, RPL5, RPL8, RPLP0, RPLP1, RPLP2, RPS10, RPS11, RPS12, RPS14, RPS15, RPS17, RPS19, RPS2, RPS20, RPS23, RPS24, RPS25, RPS27, RPS27A, RPS28, RPS3, RPS5, RPS7, RPS8, RPS9, RRP1B, SF3B1, SNRNP40, SNRPG, UBA52] |
| GO:0005840 | ribosome | GO_CellularComponent-_11.01.2018_00h00 | 0.00 | 0.00 | 0.00 | 0.00 | [4, 5, 6, 7] | Group21 | 22.48 | 49.00 | [FAU, RACK1, RPL10, RPL11, RPL13, RPL13A, RPL14, RPL18, RPL19, RPL23, RPL26, RPL27A, RPL3, RPL30, RPL31, RPL32, RPL34, RPL35, RPL35A, RPL36AL, RPL37, RPL37A, RPL38, RPL5, RPL8, RPLP0, RPLP1, RPLP2, RPS10, RPS11, RPS12, RPS14, RPS15, RPS17, RPS19, RPS2, RPS20, RPS23, RPS24, RPS25, RPS27, RPS27A, RPS28, RPS3, RPS5, RPS7, RPS8, RPS9, UBA52] |
| GO:0044391 | ribosomal subunit | GO_CellularComponent-_11.01.2018_00h00 | 0.00 | 0.00 | 0.00 | 0.00 | [3, 4, 5, 6, 7, 8] | Group21 | 25.00 | 45.00 | [FAU, RPL10, RPL11, RPL13, RPL13A, RPL14, RPL18, RPL19, RPL23, RPL26, RPL27A, RPL3, RPL30, RPL31, RPL32, RPL34, RPL35, RPL35A, RPL37, RPL37A, RPL38, RPL5, RPL8, RPLP0, RPLP1, RPLP2, RPS10, RPS11, RPS12, RPS15, RPS17, RPS19, RPS2, RPS20, RPS23, RPS24, RPS27, RPS27A, RPS28, RPS3, RPS5, RPS7, RPS8, RPS9, UBA52] |
| GO:0044445 | cytosolic part | GO_CellularComponent-_11.01.2018_00h00 | 0.00 | 0.00 | 0.00 | 0.00 | [4, 5, 6, 7, 8] | Group21 | 21.03 | 45.00 | [FAU, RPL10, RPL11, RPL13, RPL13A, RPL14, RPL18, RPL19, RPL23, RPL26, RPL27A, RPL3, RPL30, RPL31, RPL32, RPL34, RPL35, RPL35A, RPL37, RPL37A, RPL38, RPL5, RPL8, RPLP0, RPLP1, RPLP2, RPS10, RPS11, RPS12, RPS15, RPS17, RPS19, RPS2, RPS20, RPS23, RPS24, RPS27, RPS27A, RPS28, RPS3, RPS5, RPS7, RPS8, RPS9, UBA52] |
| GO:0015934 | large ribosomal subunit | GO_CellularComponent-_11.01.2018_00h00 | 0.00 | 0.00 | 0.00 | 0.00 | [4, 5, 6, 7, 8, 9] | Group21 | 22.32 | 25.00 | [RPL10, RPL11, RPL13, RPL13A, RPL14, RPL18, RPL19, RPL23, RPL26, RPL27A, RPL3, RPL30, RPL31, RPL32, RPL34, RPL35, RPL35A, RPL37, RPL37A, RPL38, RPL5, RPL8, RPLP0, RPLP1, RPLP2] |
| GO:0015935 | small ribosomal subunit | GO_CellularComponent-_11.01.2018_00h00 | 0.00 | 0.00 | 0.00 | 0.00 | [4, 5, 6, 7, 8, 9] | Group21 | 28.99 | 20.00 | [FAU, RPS10, RPS11, RPS12, RPS15, RPS17, RPS19, RPS2, RPS20, RPS23, RPS24, RPS27, RPS27A, RPS28, RPS3, RPS5, RPS7, RPS8, RPS9, UBA52] |
| GO:0022626 | cytosolic ribosome | GO_CellularComponent-_11.01.2018_00h00 | 0.00 | 0.00 | 0.00 | 0.00 | [5, 6, 7, 8, 9] | Group21 | 43.27 | 45.00 | [FAU, RPL10, RPL11, RPL13, RPL13A, RPL14, RPL18, RPL19, RPL23, RPL26, RPL27A, RPL3, RPL30, RPL31, RPL32, RPL34, RPL35, RPL35A, RPL37, RPL37A, RPL38, RPL5, RPL8, RPLP0, RPLP1, RPLP2, RPS10, RPS11, RPS12, RPS15, RPS17, RPS19, RPS2, RPS20, RPS23, RPS24, RPS27, RPS27A, RPS28, RPS3, RPS5, RPS7, RPS8, RPS9, UBA52] |
| GO:0022625 | cytosolic large ribosomal subunit | GO_CellularComponent-_11.01.2018_00h00 | 0.00 | 0.00 | 0.00 | 0.00 | [5, 6, 7, 8, 9, 10] | Group21 | 43.10 | 25.00 | [RPL10, RPL11, RPL13, RPL13A, RPL14, RPL18, RPL19, RPL23, RPL26, RPL27A, RPL3, RPL30, RPL31, RPL32, RPL34, RPL35, RPL35A, RPL37, RPL37A, RPL38, RPL5, RPL8, RPLP0, RPLP1, RPLP2] |
| GO:0022627 | cytosolic small ribosomal subunit | GO_CellularComponent-_11.01.2018_00h00 | 0.00 | 0.00 | 0.00 | 0.00 | [5, 6, 7, 8, 9, 10] | Group21 | 48.78 | 20.00 | [FAU, RPS10, RPS11, RPS12, RPS15, RPS17, RPS19, RPS2, RPS20, RPS23, RPS24, RPS27, RPS27A, RPS28, RPS3, RPS5, RPS7, RPS8, RPS9, UBA52] |
| GO:0019843 | rRNA binding | GO_MolecularFunction-_11.01.2018_00h00 | 0.00 | 0.00 | 0.00 | 0.00 | [5] | Group03 | 18.37 | 9.00 | [RPL11, RPL23, RPL37, RPL5, RPL8, RPS11, RPS3, RPS5, RPS9] |
| GO:0033613 | activating transcription factor binding | GO_MolecularFunction-_11.01.2018_00h00 | 0.03 | 0.41 | 0.03 | 0.19 | [4] | Group05 | 5.26 | 3.00 | [EGR2, NHLH2, PTMA] |
| GO:0070063 | RNA polymerase binding | GO_MolecularFunction-_11.01.2018_00h00 | 0.00 | 0.02 | 0.00 | 0.00 | [4] | Group07 | 11.36 | 5.00 | [BIN1, GSG1, PABPN1, RTF1, TAF10] |
| GO:0019209 | kinase activator activity | GO_MolecularFunction-_11.01.2018_00h00 | 0.04 | 0.36 | 0.04 | 0.14 | [4] | Group08 | 5.00 | 3.00 | [HMGB1, RPLP1, SPRY2] |
| GO:0030295 | protein kinase activator activity | GO_MolecularFunction-_11.01.2018_00h00 | 0.03 | 0.55 | 0.04 | 0.14 | [5] | Group08 | 5.56 | 3.00 | [HMGB1, RPLP1, SPRY2] |
| GO:0008135 | translation factor activity, RNA binding | GO_MolecularFunction-_11.01.2018_00h00 | 0.00 | 0.15 | 0.00 | 0.00 | [5] | Group11 | 6.76 | 5.00 | [EEF1A1, EEF1G, EIF1, EIF4A1, ELOB] |
| GO:0003746 | translation elongation factor activity | GO_MolecularFunction-_11.01.2018_00h00 | 0.00 | 0.06 | 0.00 | 0.00 | [6] | Group11 | 20.00 | 3.00 | [EEF1A1, EEF1G, ELOB] |
| GO:0003697 | single-stranded DNA binding | GO_MolecularFunction-_11.01.2018_00h00 | 0.02 | 0.51 | 0.00 | 0.00 | [5] | Group12 | 5.00 | 4.00 | [HMGB1, HNRNPA2B1, PCBP1, RTF1] |
| GO:0003729 | mRNA binding | GO_MolecularFunction-_11.01.2018_00h00 | 0.00 | 0.00 | 0.00 | 0.00 | [5] | Group12 | 6.10 | 10.00 | [DDX5, HNRNPA2B1, PCBP1, RPL13A, RPL26, RPL35, RPS3, RPS5, RPS7, SF3B1] |
| GO:0003730 | mRNA 3'-UTR binding | GO_MolecularFunction-_11.01.2018_00h00 | 0.00 | 0.19 | 0.00 | 0.00 | [6] | Group12 | 8.33 | 4.00 | [DDX5, HNRNPA2B1, PCBP1, RPS7] |
| GO:0016651 | oxidoreductase activity, acting on NAD(P)H | GO_MolecularFunction-_11.01.2018_00h00 | 0.00 | 0.03 | 0.01 | 0.07 | [3] | Group19 | 7.69 | 6.00 | [ND1, ND2, ND3, ND4, ND5, ND6] |
| GO:0003954 | NADH dehydrogenase activity | GO_MolecularFunction-_11.01.2018_00h00 | 0.00 | 0.00 | 0.01 | 0.07 | [4] | Group19 | 19.35 | 6.00 | [ND1, ND2, ND3, ND4, ND5, ND6] |
| GO:0016655 | oxidoreductase activity, acting on NAD(P)H, quinone or similar compound as acceptor | GO_MolecularFunction-_11.01.2018_00h00 | 0.00 | 0.00 | 0.01 | 0.07 | [4] | Group19 | 17.65 | 6.00 | [ND1, ND2, ND3, ND4, ND5, ND6] |
| GO:0050136 | NADH dehydrogenase (quinone) activity | GO_MolecularFunction-_11.01.2018_00h00 | 0.00 | 0.00 | 0.01 | 0.07 | [5] | Group19 | 22.22 | 6.00 | [ND1, ND2, ND3, ND4, ND5, ND6] |
| GO:0008137 | NADH dehydrogenase (ubiquinone) activity | GO_MolecularFunction-_11.01.2018_00h00 | 0.00 | 0.00 | 0.01 | 0.07 | [6] | Group19 | 22.22 | 6.00 | [ND1, ND2, ND3, ND4, ND5, ND6] |
| GO:0003723 | RNA binding | GO_MolecularFunction-_11.01.2018_00h00 | 0.00 | 0.00 | 0.00 | 0.00 | [4] | Group21 | 4.63 | 62.00 | [DDX5, DNAJC2, EEF1A1, EEF1G, EIF1, EIF4A1, ELOB, FAU, HMGB1, HNRNPA0, HNRNPA2B1, LSM2, PABPN1, PCBP1, PDAP1, PSMA6, RACK1, RBBP6, RPL10, RPL11, RPL13, RPL13A, RPL14, RPL18, RPL19, RPL23, RPL26, RPL27A, RPL3, RPL30, RPL32, RPL34, RPL35, RPL35A, RPL37, RPL37A, RPL38, RPL5, RPL8, RPLP0, RPS10, RPS11, RPS15, RPS17, RPS2, RPS20, RPS23, RPS27A, RPS28, RPS3, RPS5, RPS7, RPS8, RPS9, RRP1B, RTF1, SF3B1, SNRNP40, SNRPG, SSRP1, UBA52, YWHAZ] |
| GO:0055106 | ubiquitin-protein transferase regulator activity | GO_MolecularFunction-_11.01.2018_00h00 | 0.00 | 0.07 | 0.00 | 0.00 | [3] | Group22 | 18.75 | 3.00 | [RPL11, RPS7, TRIB2] |
| GO:0003729 | mRNA binding | GO_MolecularFunction-_11.01.2018_00h00 | 0.00 | 0.00 | 0.00 | 0.00 | [5] | Group22 | 6.10 | 10.00 | [DDX5, HNRNPA2B1, PCBP1, RPL13A, RPL26, RPL35, RPS3, RPS5, RPS7, SF3B1] |
| KEGG:04722 | Neurotrophin signaling pathway | KEGG_11.01.2018 | 0.02 | 0.50 | 0.02 | 0.14 | [-1] | Group00 | 4.03 | 5.00 | [ARHGDIB, CALM1, CAMK2D, NFKBIA, NGF] |
| KEGG:05134 | Legionellosis | KEGG_11.01.2018 | 0.03 | 0.39 | 0.00 | 0.00 | [-1] | Group11 | 5.17 | 3.00 | [EEF1A1, EEF1G, NFKBIA] |
| KEGG:03040 | Spliceosome | KEGG_11.01.2018 | 0.00 | 0.10 | 0.00 | 0.03 | [-1] | Group13 | 5.19 | 7.00 | [DDX5, LOC785761, LSM2, PCBP1, SF3B1, SNRNP40, SNRPG] |
| KEGG:04666 | Fc gamma R-mediated phagocytosis | KEGG_11.01.2018 | 0.00 | 0.32 | 0.00 | 0.01 | [-1] | Group18 | 5.49 | 5.00 | [ARF6, ARPC2, BIN1, CFL1, MARCKS] |
| KEGG:00190 | Oxidative phosphorylation | KEGG_11.01.2018 | 0.00 | 0.01 | 0.01 | 0.07 | [-1] | Group19 | 6.38 | 9.00 | [ATP6, COX1, LOC101902937, ND1, ND2, ND3, ND4, ND5, ND6] |
| KEGG:05012 | Parkinson's disease | KEGG_11.01.2018 | 0.00 | 0.00 | 0.01 | 0.07 | [-1] | Group19 | 6.62 | 10.00 | [ATP6, COX1, LOC101902937, ND1, ND2, ND3, ND4, ND5, ND6, UBB] |
| KEGG:03010 | Ribosome | KEGG_11.01.2018 | 0.00 | 0.00 | 0.00 | 0.00 | [-1] | Group21 | 29.52 | 49.00 | [FAU, LOC787803, RPL10, RPL11, RPL13, RPL13A, RPL14, RPL18, RPL19, RPL23, RPL26, RPL27A, RPL3, RPL30, RPL31, RPL32, RPL34, RPL35, RPL35A, RPL36AL, RPL37, RPL37A, RPL38, RPL5, RPL8, RPLP0, RPLP1, RPLP2, RPS10, RPS11, RPS12, RPS14, RPS15, RPS17, RPS19, RPS2, RPS20, RPS23, RPS24, RPS25, RPS27, RPS27A, RPS28, RPS3, RPS5, RPS7, RPS8, RPS9, UBA52] |
